# Supplementary material for: Repetitive Transcranial Magnetic Stimulation targeted with MRI based neuro-navigation in major depressive episode: a double-blind, multicenter randomized controlled trial
Source: PLoS One. 2025 May 27;20(5):e0317597. doi: 10.1371/journal.pone.0317597 (PMC12111610; doi:10.1371/journal.pone.0317597)
Supplement: S1 File — (DOC) [file pone.0317597.s001.doc]

| PROMOTEUR : | **CHRU DE RENNES**  **2, rue Henri Le Guilloux**  **35033 Rennes cedex 9** | |
| --- | --- | --- |
| **PROTOCOLE D’ESSAI CLINIQUE**  ***(TMS dépression)*** | | |
| CODE ESSAI | LOC 1021 Pr Millet Neuronavigateur | |
| N° EudraCT | 2011-A01272-39 | |
| DISPOSITIF MEDICAL |  | |
| TITRE COMPLET | Evaluation de la stimulation magnétique transcrânienne répétée (rTMS) assistée d'un système de neuronavigation dans le traitement des états dépressifs caractérisés | |
| PHASE CLINIQUE | Essai randomisé, en double aveugle, contrôlé, multicentrique | |
| INDICATION(S) (CIBLE) | *Traitement Etats dépressifs caractérisés* | |
| INVESTIGATEURS PRINCIPAUX | Pr Dominique Drapier Pr Bruno Millet | |
|  | Centre Hospitalier Guillaume Regnier  108 AV. DU GENERAL LECLERC  - B.P. 60321  35703 Rennes | .Groupe Hospitalier Pitié-Salpêtrière, Service de Psychiatrie Adulte, Pavillon La Force,  47 boulevard de l'Hôpital,  75651 Paris Cedex 13. |
| N° DE VERSION DU PROTOCOLE | *Version 12.0* | |
| DATE DU PROTOCOLE | *Le 14 septembre 2015* | |
| CPP | *Approuvé le 20 mars 2012*  *Par le Comité de Protection des Personnes de Brest Ouest VI* | |
| AFSSAPS | *Date d’autorisation : 30 janvier 2012*  *N° d’autorisation : 2011-A01272-39 (ref.UEC/DA/2012-035)* | |
| **CE DOCUMENT CONFIDENTIEL EST LA PROPRIETE DU CHRU DE RENNES**  **AUCUNE INFORMATION NON PUBLIEE FIGURANT DANS CE DOCUMENT NE PEUT ETRE DIVULGUEE SANS AUTORISATION ECRITE PREALABLE DU CHRU DE RENNES** | | |

Version précédente diffusée : Version 10.0 du 30 janvier 2015 (modification substantielle n° 5)

Version soumise aux instances : Version 11.0 du 17 juillet 2015 (modification substantielle n°6)

Version diffusée : version 12.0 du 14 septembre 2015

Investigateurs Coordonnateurs : Pr Dominique Drapier et Pr Bruno Millet, PU-PH

URU (Comportements et Noyaux Gris Centraux), Université de Rennes 1

Promoteur : Centre Hospitalier Universitaire de Rennes

Partenariat : Société SYNEIKA

SIGNATURES

SIGNATURE DE L'INVESTIGATEUR

| J'ai lu l’ensemble des pages du protocole de l’essai clinique dont le CHRU de Rennes est le promoteur. Je confirme qu'il contient toutes les informations nécessaires à la conduite de l’essai. Je m'engage à réaliser l’essai en respectant le protocole et les termes et conditions qui y sont définis. Je m'engage à réaliser l’essai en respectant :   - les principes de la “Déclaration d’Helsinki”, - les règles et recommandations de bonnes pratiques cliniques internationales (ICH-E6) et française (règles de bonnes pratiques cliniques pour les recherches biomédicales portant sur des dispositifs à usage humain - décisions du 24 novembre 2006) - la législation nationale et la réglementation relative aux essais cliniques, - la conformité avec la Directive Essais Cliniques de l’UE [2001/20/EC] dont une copie de chaque m'a été remise par le promoteur.   Je m'engage également à ce que les investigateurs et les autres membres qualifiés de mon équipe aient accès aux copies de ce protocole et des documents relatifs à la conduite de l’essai pour leur permettre de travailler dans le respect des dispositions figurant dans ces documents. | |
| --- | --- |
| **NOM: Pr Dominique Drapier**  Signature : …………………………………………….. | Date : ___________________ |

SIGNATURE DU PROMOTEUR

| **Promoteur : CHU de Rennes** | |
| --- | --- |
| NOM : Yves Rayer  Signature : ……………………………………………… | Date : ___________________ |

# SOMMAIRE

[1 JUSTIFICATION DE L’ETUDE 10](#__RefHeading___Toc430002373)

[1.1 Introduction 10](#__RefHeading___Toc430002374)

[1.2 La Stimulation Magnétique Transcrânienne répétitive 10](#__RefHeading___Toc430002375)

[1.3 Traitement de la dépression par la rTMS 12](#__RefHeading___Toc430002376)

[1.4 Le problème de la localisation 14](#__RefHeading___Toc430002377)

[1.5 Conclusion 16](#__RefHeading___Toc430002378)

[2 OBJECTIFS 16](#__RefHeading___Toc430002379)

[2.1 Objectif principal 16](#__RefHeading___Toc430002380)

[2.2 Objectifs secondaires 16](#__RefHeading___Toc430002381)

[3 SYSTEME DE NEURONAVIGATION 16](#__RefHeading___Toc430002382)

[3.1 Principe de fonctionnement 16](#__RefHeading___Toc430002383)

[4 SCHEMA DE L’ETUDE 18](#__RefHeading___Toc430002384)

[5 POPULATION ETUDIEE 18](#__RefHeading___Toc430002385)

[5.1 Critères d’inclusion 18](#__RefHeading___Toc430002386)

[5.2 Critères de non inclusion 19](#__RefHeading___Toc430002387)

[6 RANDOMISATION 19](#__RefHeading___Toc430002388)

[6.1 Constitution de la liste de randomisation 19](#__RefHeading___Toc430002389)

[7 DEROULEMENT DE L’ETUDE 20](#__RefHeading___Toc430002390)

[7.1 Phase d’éligibilité 20](#__RefHeading___Toc430002391)

[7.2 Phase d’inclusion et suivi thérapeutique des patients 20](#__RefHeading___Toc430002392)

[7.3 Protocole thérapeutique de la rTMS et neuronavigation 21](#__RefHeading___Toc430002393)

[8 PARAMETRES ETUDIES 23](#__RefHeading___Toc430002394)

[8.1 Description des outils 23](#__RefHeading___Toc430002395)

[8.2 Paramètres évalués 24](#__RefHeading___Toc430002396)

[9 CRITERES DE JUGEMENT 25](#__RefHeading___Toc430002397)

[9.1 Critère de jugement principal 25](#__RefHeading___Toc430002398)

[9.2 Critères de jugement secondaire 25](#__RefHeading___Toc430002399)

[10 NOMBRE DE SUJETS NECESSAIRE 25](#__RefHeading___Toc430002400)

[11 ANALYSE STATISTIQUE 25](#__RefHeading___Toc430002401)

[11.1 Analyse descriptive 26](#__RefHeading___Toc430002402)

[11.2 Comparaison des groupes en fonction du traitement reçu 26](#__RefHeading___Toc430002403)

[11.3 Analyse des évènements indésirables 26](#__RefHeading___Toc430002404)

[12 GESTION DES EVENEMENTS INDESIRABLES 26](#__RefHeading___Toc430002405)

[12.1 Définitions 26](#__RefHeading___Toc430002406)

[12.2 Rôle de l’investigateur 27](#__RefHeading___Toc430002407)

[12.3 Rôle du promoteur 28](#__RefHeading___Toc430002408)

[13 PERDUS DE VUE ET SORTIE D’ESSAI 28](#__RefHeading___Toc430002409)

[13.1 Procédure de gestion des perdus de vue 28](#__RefHeading___Toc430002410)

[13.2 Procédure de gestion des sorties d’essai 28](#__RefHeading___Toc430002411)

[14 SURVEILLANCE DE L’ESSAI 29](#__RefHeading___Toc430002412)

[14.1 Centre de Coordination de l’étude 29](#__RefHeading___Toc430002413)

[14.2 Comité de pilotage 29](#__RefHeading___Toc430002414)

[14.3 Comité de Validation des Données 29](#__RefHeading___Toc430002415)

[15 ASPECTS LOGISTIQUES, LEGAUX ET GENERAUX 29](#__RefHeading___Toc430002416)

[15.1 Calendrier prévisionnel 29](#__RefHeading___Toc430002417)

[15.2 Faisabilité de l’étude 29](#__RefHeading___Toc430002418)

[15.3 Page de signature du protocole 30](#__RefHeading___Toc430002419)

[15.4 Soumission du protocole au CCP et déclaration à l’autorité compétente 30](#__RefHeading___Toc430002420)

[15.5 Amendement au protocole 30](#__RefHeading___Toc430002421)

[15.6 Notice d’information au patient et consentement 30](#__RefHeading___Toc430002422)

[15.7 Transcription des données et archivage 31](#__RefHeading___Toc430002423)

[15.8 Saisie des données 31](#__RefHeading___Toc430002424)

[15.9 Responsabilité 31](#__RefHeading___Toc430002425)

[15.10 Rapports fournis en cours et en fin d’étude 31](#__RefHeading___Toc430002426)

[15.11 REGLES DE PUBLICATION 31](#__RefHeading___Toc430002427)

[16 LE BUDGET 32](#__RefHeading___Toc430002428)

[References bibliographiques 33](#__RefHeading___Toc430002429)

[17 ANNEXES 37](#__RefHeading___Toc430002430)

[17.1 ANNEXE 1 : FORMULAIRE D’INFORMATION 37](#__RefHeading___Toc430002431)

[17.2 ANNEXE 2 : Formulaire de consentement 38](#__RefHeading___Toc430002432)

[17.3 ANNEXE 3 39](#__RefHeading___Toc430002433)

[17.4 ANNEXE 4 40](#__RefHeading___Toc430002434)

Résumé de la recherche

| Titre | **Evaluation de la stimulation magnétique transcrânienne répétée (rTMS) assistée d'un système de neuronavigation dans le traitement des états dépressifs caractérisés** |
| --- | --- |
| Promoteur | CHU de Rennes |
| Investigateurs Coordinateurs | Pr Dominique Drapier- Centre Hospitalier Guillaume Regnier  108 AV. DU GENERAL LECLERC - B.P. 60321 35703 Rennes  Et  Pr Bruno Millet Groupe Hospitalier Pitié-Salpêtrière, Service de Psychiatrie Adulte, Pavillon La Force,  47 boulevard de l'Hôpital,  75651 Paris Cedex 13. |
| Version du protocole | version 12.0 du 14 septembre 2015 |
| Justification / contexte | La Stimulation Magnétique Transcrânienne répétée (rTMS) est un nouvel outil venant compléter l’éventail des traitements contre la dépression. La rTMS est une technique permettant de stimuler le tissu cérébral de manière localisée et non invasive. Un courant électrique bref traverse une bobine pour créer un champ magnétique transitoire qui lui-même induit un champ électrique au travers des tissus conducteurs. Ce champ électrique peut modifier le potentiel trans-synaptique des neurones situés directement sous le faisceau de la bobine.  Plusieurs méta-analyses (Gershon & al, 2003 ; Loo & al, 2005 ; Couturier & al, 2005) ont montré la supériorité de la rTMS par rapport au placebo bien que ces résultats soient tempérés par le faible échantillon de patients par étude et l’hétérogénéité des méthodologies utilisées. En outre, quelques études (Rumi & al, 2005) montrent que cette technique permet d’accélérer la réponse aux antidépresseurs classiques. Cette technique vient par ailleurs d’avoir l’accord de la Food and Drug Administration (FDA) pour être utilisée dans un cadre de routine clinique dans l’indication de la dépression.  Les paramètres de stimulations sont nombreux (fréquence, intensité, nombre de séances, nombre de pulses par séance) et leurs effets commencent à être mieux définis afin d’être prédictifs d’une meilleure efficacité (Gross & al, 2007) tout en respectant des critères de sécurité (Gershon & al, 2003 ; Wasserman, 1996 ; Chen & al, 1997).  Cependant, la cible de stimulation, le Cortex Pré Frontal Dorsolatéral (CPFDL) et sa méthode de détermination décrite par Georges & al (1995) n’ont pas évolué. Cette méthode, basée sur un repérage anatomique ne prend pas en compte les variations anatomiques interindividuelles (Herwig & al, 2001) et reste opérateur dépendant (Mémoire de master 2 C. Nauczyciel). Dans la mesure où le CPFDL est la cible privilégiée de la rTMS dans la dépression, l’imprécision de son repérage peut avoir des conséquences thérapeutiques qui nécessitent une évaluation. Fitzgerald & al (2009) a ainsi montré sur un faible échantillon de patients qu’un repérage précis et reproductible du CPFDL permettait une amélioration thérapeutique supérieure par rapport au repérage standard. Cette étude nécessite d’être améliorée, en particulier avec un échantillon suffisant de patients. |
| Objectif Principal | Au vues des données de la littérature, l’objectif de ce projet est d’évaluer par une étude multicentrique randomisée en double aveugle sur deux groupes parallèles, la supériorité thérapeutique de la stimulation localisée du cortex préfrontal dorsolatéral (CPFDL) gauche par un neuronavigateur par rapport à la stimulation du cortex préfrontal dorsolatéral (CPFDL) gauche repéré de manière standard dans le traitement des états dépressifs isolés ou récurrents.  Notre hypothèse est qu’une stimulation localisée du CPFDL permet une réponse clinique qualitativement et quantitativement supérieure à une stimulation moins précise. |
| Objectifs Secondaires | Tester la supériorité du ressenti de l’amélioration thérapeutique chez les patients stimulés avec le système de neuronavigation par rapport aux patients stimulés avec la méthode standard au moyen du Beck Depression Inventory (BDI)  Tester la supériorité de l’amélioration du plan psychomoteur chez les patients stimulés avec le système de neuronavigation par rapport aux patients stimulés avec la méthode standard au moyen de l’Echelle de Dépression et de Ralentissement (EDR) |
| Critère de Jugement Principal | Le critère de jugement principal est l’obtention d’une réponse clinique définie par une diminution de la moitié du score de la MADRS à J44, à l’issue du traitement par TMS  et de la période de suivi. |
| Critères de Jugement Secondaires | Un des critères de jugement secondaire est le pourcentage de patients présentant une rémission clinique à J14 et J44.  La rémission clinique est définie par un score à la MADRS inférieur ou égal à 8.  Le pourcentage de patients répondeurs à J14 est également un critère secondaire.  Le ressenti de l’amélioration clinique est également évalué via la variation de l’échelle BDI après le traitement à J14 et au cours du suivi (J44) dans chaque groupe.  Le ralentissement psychomoteur est évalué via la variation de l’échelle ERD après le traitement et au cours du suivi dans chaque groupe. |
| Méthodologie / Schéma de l’etude | Etude multicentrique randomisée en double aveugle de patients répondant aux critères diagnostiques d’Episode dépressif Majeur isolé ou récurrent, répartis en :   - un groupe de 60 patients bénéficiant d’un repérage anatomique empirique de la cible et d’une stimulation non assistée par un neuronavigateur - un groupe de 60 patients bénéficiant d’un repérage de la cible en IRM et d’une stimulation guidée par neuronavigateur.   Les paramètres de stimulation utilisés seront les suivants: 10 séances, 20Hz, 110 % du seuil moteur, 1600 pulses/séance.  *Critère principal d’évaluation* : amélioration du score à la Montgomery and Adsberg Rating Scale (MADRS ; Montgomery et al, 1979) avant et après la stimulation magnétique transcrânienne ainsi que 30 jours après la fin des stimulations.  *Critères secondaires d’évaluation* : variations des scores aux échelles de Beck Depression Inventory (BDI) et à l’Echelle de Ralentissement (EDR).  Les patients sont évalués par un cotateur avant la première séance, après les 10 séances et un mois après la fin des séances par ce même cotateur. Les séances de stimulations sont réalisées par un opérateur indépendant du cotateur.  Les centres investigateurs impliqués seront les suivants : CHU de Rennes/CHGR, CHU de Brest, EPSM Saint-Avé, CESAME Angers, EPSM Quimperlé, Clinique St Laurent (Rennes). La durée de recrutement des patients est fixée à 2 ans.  Cette étude sera réalisée après avis du CPP dans le respect des personnes (information, consentement éclairé et confidentialité). Elle sera réalisée conformément aux règles de bonnes pratiques cliniques. |
| Critères d’Inclusion des Sujets | Sujets volontaires droitiers de plus de 18 ans et de moins de 65 ans :   - Ayant signé un consentement libre et éclairé à la participation à l’étude. - Répondant au diagnostic d’épisode dépressif majeur (EDM) récurrent ou isolé selon les critères du DSM IV (A.P.A. 1994). - Ayant un traitement antidépresseur non modifié depuis 3 semaines - Score à l’échelle MADRS ≥ 21   Le traitement antidépresseur des patients ne doit pas être modifié dans les 3 semaines précédant l’inclusion pour éviter les biais de confusion entre traitement antidépresseur et traitement par rTMS. Les traitements par benzodiazépines doivent être arrêtés dans la mesure où ils peuvent être substitués par d’autres traitements anxiolytiques et qu’ils diminuent l’excitabilité corticale. Les traitements thymorégulateurs, bien qu’ils diminuent également l’excitabilité corticale ne sont pas arrêtés car ils ne peuvent être substitués. |
| Critères de Non-Inclusion des Sujets | - Dépression avec caractéristiques psychotiques - Diagnostic co-morbide selon l’axe I (DSM IV) de schizophrénie, de dépendance (ou d’abus) à l’alcool et/ou à une autre substance (vie entière) - Patient répondant aux critères de la dépression résistante définie par le stade V de la classification de Thase & Rush (voir annexe 4) - Patient hospitalisé sous contrainte ou sous mesure de protection juridique (tutelle, curatelle) - Patient présentant un risque suicidaire élevé (item 10 MADRS > 3) en l’absence d’une hospitalisation - Contre-indication à la pratique de l’IRM ou de la rTMS : antécédents personnels de crise comitiale, de pathologies neurologiques ou neurochirurgicales, matériel prothétique métallique ou corps étrangers (pacemaker, matériel ferromagnétique intra oculaire) - Patient de plus de 65 ans en raison du risque d’atrophie corticale. - Grossesse. |
| DISPOSITIFS/ Stratégies / Procédures | Une stimulation localisée du CPFDL gauche par un neuronavigateur est comparée à la stimulation du CPFDL gauche repéré de manière standard dans le traitement des états dépressifs isolés ou récurrents.  Quelque soit le bras dans lequel ont été randomisés les patients, tous doivent avoir passé une IRM 3D nécessaire à la neuronavigation.  - Si un patient a déjà bénéficié au cours de son suivi habituel d'une IRM 3D alors celle-ci sera utilisée sans en refaire ;  - Si un patient n'a jamais bénéficié d'une IRM 3D, alors celle-ci devra être réalisée dans les 15 jours suivant la randomisation."  Les patients, après avoir été randomisés, sont affectés à un des traitements suivants :  - rTMS à haute fréquence (20Hz) appliquée au niveau du CPFDL gauche repéré par neuronavigation  - rTMS à haute fréquence (20Hz) appliquée au niveau du CPFDL gauche repéré par la méthode standard dite « des 5 cm »  La première séance de TMS doit être effectuée au plus tard 15 jours après la randomisation. Elle définit le J0 de l’étude.  Le traitement est délivré par un clinicien différent de l’investigateur-cotateur, informé par le CIC du bras de randomisation.  Les échelles MADRS, BDI et ERD sont réalisées par l’investigateur-cotateur aveugle au bras de traitement, avant la première séance de TMS.  Après les 10 séances de traitement par rTMS, les patients entrent dans une phase de suivi au cours duquel ils sont évalués 2 fois : après la dernière séance (J14) et 1 mois après la dernière séance (J44).  Le traitement antidépresseur, stable depuis au moins 3 semaines ne sera modifié qu’en cas d‘urgence thérapeutique. Toute modification sera reportée dans le cahier d’observation.  En cas de risque suicidaire majeur (item 10 MADRS> 3), les patients seront hospitalisés conformément aux bonnes pratiques cliniques.  **Schéma du protocole :**  INCLUSION  10 SEANCES TMS  SUIVI DES PATIENTS  Absence de CI  IRM  Neuronavigation ou méthode des 5cm  Echelles : MADRS, BDI, EDR  J0  J14  J44 |
| Nombre de Patients | La question posée est en formulation bilatérale.  Le taux attendu de patients améliorés (MADRS diminuée de 50%) dans le bras « stimulation standard » est de 35%. Le bénéfice attendu avec la neuronavigation doit permettre d’augmenter ce taux à 70% Si l’on veut se donner les moyens statistiques de détecter une augmentation absolue de 35% dans le bras « neuronavigation » il faut inclure 60 sujets par bras pour garantir une puissance de 95% dans un test réalisé avec un risque d’erreur α = 5%.  Le nombre total de sujets nécessaires est donc de **120**. |
| Durée de la Recherche | Date de début : janvier 2013  Période de recrutement : 4 ans  Durée de suivi : 59 jours  Durée totale de l’étude : 52 mois  Date de fin d'étude : avril 2017 |
| Retombées attendues | *Retombées attendues de l’étude*  Nous faisons l’hypothèse d’une amélioration qualitativement et quantitativement supérieure des patients stimulés avec le système de neuronavigation par rapport aux patients stimulés par la méthode standard.  Perspectives : la validation de l’intérêt de la stimulation assistée d’un neuronavigateur permettra d’affiner le repérage du CPFDL en couplant neuronavigation et imagerie fonctionnelle, d’évaluer l’intérêt de nouvelles cibles dans la dépression actuellement non accessibles à un repérage manuel |

# JUSTIFICATION DE L’ETUDE

## Introduction

La dépression est une des pathologies chroniques les plus fréquentes dans le monde avec 121 millions de personnes atteintes selon l’OMS. Selon cette source, les troubles dépressifs représentent la 1ière cause de morbidité avec des répercussions sur le plan humain, fonctionnel et économique.

Actuellement, la prise en charge d’un épisode dépressif repose principalement sur les antidépresseurs associés à une psychothérapie de soutien. Néanmoins, les traitements médicamenteux présentent des limites : plus de 15 % des dépressions sont/deviennent résistantes (Fava & al, 2003). Il existe d’autres techniques tels que les sismothérapies permettant un meilleur résultat thérapeutique (Geddes & al, 2003) mais au prix d’une anesthésie et d’effets secondaires invalidants tels que les troubles mnésiques (Lisanby & al, 2000). Il est donc important de développer des techniques alternatives de traitement de la dépression. La stimulation magnétique transcrânienne répétitive (rTMS) appartient à ce nouvel ensemble de techniques de prise en charge des troubles dépressifs.

## La Stimulation Magnétique Transcrânienne répétitive

### PRINCIPE

La stimulation magnétique transcrânienne (TMS) est une technique permettant de stimuler le tissu cérébral de manière localisée et non invasive.

Elle repose sur le principe de l’induction mutuelle électromagnétique (se manifestant par un potentiel électrique apparaissant aux bouts d’un conducteur se déplaçant dans un champ magnétique) décrit par Michel Faraday.

Dans la TMS, un courant électrique bref traverse une bobine pour créer un champ magnétique transitoire qui lui-même induit un champ électrique pouvant alors passer au travers des tissus conducteurs. Il peut donc modifier le potentiel trans-synaptique des neurones situés directement sous le faisceau de la bobine. Si ce potentiel est suffisamment important (effet de seuil), il entraîne une dépolarisation neuronale et un potentiel d’action se propageant le long des axones et se transmettant par l’intermédiaire des synapses. Son intensité diminue au fur et à mesure que l’on s’éloigne de la zone stimulée.

La forme du champ magnétique et son point focal dépendent de la forme de la bobine induisant le champ électromagnétique. Il existe plusieurs formes de bobines utilisées : circulaire (champ magnétique large et diffus) ou en huit (deux bobines circulaires côte à côte donnant naissance à un champ magnétique focal, perpendiculaire au point de contact des 2 bobines mais de décroissance rapide : voir Gershon & al, 2003).

Il existe plusieurs types de stimulation : stimulation en simple pulse ou double pulse utilisée principalement en recherche fondamentale pour les études de cartographie cérébrale ou d’excitabilité corticale et stimulation répétitive où des trains de pulses sont répétés à des fréquences variables entraînant une modification des connections synaptiques. Cette technique est utilisée dans un cadre thérapeutique.

La TMS sous sa forme actuelle a été introduite par Barker en 1985. Elle est dans un premier temps utilisée en Neurologie dans le cadre du diagnostic de trouble de la conduction motrice et dans l’évaluation fonctionnelle de lésions corticales de manière non invasive. Elle est utilisée également en recherche fondamentale dans le cadre de la cartographie cérébrale et de l’étude de l’excitabilité corticale.

### PARAMETRES DE STIMULATION

L’action physiologique de la rTMS sur une région corticale permet une activation locale de cette région stimulée et une action à distance (Nahas & al, 2001) par le biais des réseaux corticaux auxquels la région corticale stimulée appartient (Paus & al, 1997). Elle dépend d’un grand nombre de paramètres techniques et humains.

- **Intensité de stimulation**

Le degré d’excitabilité corticale conditionne la réponse corticale à une stimulation électromagnétique (dépolarisation neuronale ou hyperpolarisation neuronale). Il peut être mesuré facilement sur des zones « parlantes » comme par exemple le cortex moteur, le cortex visuel alors qu’il n’est pas mesurable sur des zones « muettes » (absence d’indicateurs cliniques).

Par habitude, on prend comme référence l’intensité minimale nécessaire à produire une réponse motrice du muscle court abducteur du pouce controlatéral à la stimulation qui n’est qu’une approximation de l’intensité de stimulation de la zone cible (impossible à définir actuellement de manière non invasive). Elle varie en fonction de nombreux paramètres tels que la distance scalp-cortex (étude de Knecht & al, 2005) ou l’existence d’une variation interindividuelle de l’excitabilité corticale (Maeda & al, 2000)

- **Fréquence de stimulation**

Les fréquences de stimulation sont de deux types :

- hautes fréquences de stimulation supérieure à 1Hz, souvent entre 5 et 20 Hz
- basses fréquences de stimulation inférieure ou égale à 1Hz

Les hautes et basses fréquences n’entraînent pas les mêmes modifications corticales. En effet, une stimulation à haute fréquence du cortex moteur primaire entraîne une augmentation de l’excitabilité corticale et favorise la dépolarisation neuronale alors qu’une stimulation à basse fréquence du cortex moteur primaire produit l’effet inverse (Pascual-Leone & al, 1994 ; Chen & al, 1997).

En fonction de l’effet recherché et du site de stimulation, on utilise les deux types de fréquences.

- **Positionnement de la bobine**

La bobine en 8 induit le plus grand courant sous son centre au point où les deux circonférences des bobines se rejoignent (Cohen & al, 1999). Cette bobine doit donc être posée tangentiellement sur le scalp afin que son centre soit en regard avec la zone cérébrale à stimuler. La taille maximale du champ induit ne dépasse pas les deux centimètres et ne permet qu’une stimulation superficielle corticale.

Si le centre de la bobine n’est pas tangent au scalp du patient, le champ induit se disperse sans pénétrer dans le tissu cérébral.

- **Facteurs humains**

L’action de la rTMS sur les structures corticales varie en fonction de l’état physique et électrique de ces structures. Ainsi une atrophie corticale entraînant de fait une augmentation de la distance scalp-cortex comme on peut le rencontrer chez les sujets âgés diminue l’efficacité de l’impact de la stimulation (Gershon & al, 2003). De la même manière, tout facteur modifiant l’excitabilité corticale (maladie neurologique, prise de psychotrope) entraîne une modification de la réponse à la TMS.

### TOLERANCE ET EFFETS SECONDAIRES

La TMS est un traitement bien toléré et comportant peu d’effets secondaires. En effet, dès 1993, Pascual-Leone & al, ont montré dans une étude sur 9 sujets sains stimulés à différents points du scalp à différentes fréquences et intensités que la technique était bien tolérée. Anderson & al, 2006, ont montré l’existence d’une tolérance élevée (nombre de pulse/jour ) chez des volontaires sains avec la tolérance sans effets secondaires de 12960 pulses/jour, 3 jours consécutifs.

Les effets secondaires à une rTMS sont soit d’ordre somatique allant des céphalées à la crise comitiale soit d’ordre psychiatrique pouvant entraîner un virage maniaque lors du traitement de la dépression. Dans une revue de la littérature, Machii & al, 2006 a montré que sur 74 études ayant un questionnaire sur la tolérance et les effets secondaires 30 ont trouvé une bonne tolérance dont 16 aucun effet secondaire et 45 ont retrouvé des effets secondaires au premier rang desquels céphalées et cervicalgies, douleur au point de stimulation, 4 crises comitiales et 3 inductions maniaques.

Les céphalées sont les effets secondaires les plus signalés. Elles sont plus fréquentes lors des stimulations à basse fréquence, frontales mais existent aussi lors des protocoles avec des « bobines placebo ». L’inconfort lié au bruit des stimulations et des douleurs cervicales sont également signalés mais plus rarement. Cependant, ces effets secondaires répondent toujours aux antalgiques de niveau I.

La crise comitiale est l’événement le plus grave pouvant survenir lors de traitement par TMS.

Selon une étude de Pascual-Leone (1993) reprise par Wassermann & al (1998) pour donner des règles de sécurité, les crises comitiales sont secondaires à différents facteurs inhérents au patient (antécédents de maladie épileptique ou de maladie du système nerveux central, antécédent de traumatisme crânien grave, antécédent de crise comitiale) ou inhérents au traitement (trains de fréquence, d’intensité et de durée trop importantes)

Afin de prévenir ces crises, Wasserman, 1998, définit des guidelines de sécurité en fonction de l’intensité et de la fréquence de stimulation. Ces règles sont affinées par les travaux de Chen & al, 1997 qui inclut dans ces règles les intervalles inter train d’onde de stimulation.

Les inductions d’état mixtes ou maniaques semblent être des événements relativement rares (Sakkas & al, 2003 ; Machii & al, 2006) et survenir en particulier chez des patients présentant un trouble bipolaire de type 1 ou 2.

Les répercussions cognitives de la rTMS sont l’objet d’études contradictoires. Li et al, 2007 ; ont retrouvé un effet délétère sur la mémoire spatiale à court et long terme chez des rats stimulés « cerveau entier » à basse fréquence pendant 10 séances. Cette étude est cependant difficilement transposable à l’homme. Chez l’homme, les études sur les conséquences cognitives de la rTMS ont donné des résultats contradictoires mais avec des protocoles de stimulation hétérogènes. Vanderhasselt et al, 2007 ont montré une augmentation des capacités attentionnelles chez des patients déprimés après une séance de stimulation du CPFDL gauche à haute fréquence. Martis & al, 2003, ont montré qu’elle améliore certains domaines mnésiques et la dextérité motrice. Aucune étude n’a démontré à notre connaissance d’effet délétère sur le plan cognitif perdurant dans les suites d’un traitement par rTMS.

## Traitement de la dépression par la rTMS

### PROPRIETES ANTIDEPRESSIVES DE LA rTMS

Les effets thymiques de la rTMS ont été décrits dès 1993 (Hoflich & al, 1993) Ces travaux se sont poursuivis avec ceux de Georges & al, 1995, 1996 et Pascual-Leone & al, 1996 avec des études montrant une modification de l’humeur induite par la rTMS et cela en fonction de la latéralisation de la stimulation. A partir de 1996, des études thérapeutiques sur la dépression résistante par la rTMS d’abord ouvertes puis randomisées avec des conditions de contrôle sont effectuées.

Les 7 méta-analyses (Holtzheimeir & al, 2001 ; McNamara & al, 2001 ; Kozel & al , 2002 ; Martin & al, 2003; Loo & Mitchell, 2005, Couturier & al, 2005 ; Herrmann & Ebmeier, 2006) montrent que les études contre placebo retrouvent une supériorité faible mais significative du traitement de la rTMS par rapport au placebo (Pascual-Leone, 1996 ; George & al ,1997 ; Eschweiler & al, 2000 ; Filzgerald & al, 2003 ; Avery & al, 2006, Padberg & George, 2009). Cependant, au fur et à mesure que ces études sont menées, les paramètres de stimulation sont affinés permettant d’améliorer l’efficacité thérapeutique du traitement (Gross & al, 2007). Ainsi pour le traitement de la dépression, une intensité de la stimulation supraliminaire permet une meilleure efficacité thérapeutique (Loo & Mitchell, 2005 ; Padberg & al, 2002 ; Nahas & al, 2001). La fréquence de stimulation varie en fonction du côté stimulé  mais avec une efficacité comparable (Filzgerald & al, 2003): haute fréquence pour le CPFDL gauche ou basse fréquence pour le CPFDL droit. Le nombre de séance et le nombre d’impulsions par séance conditionnent également l’efficacité thérapeutique : meilleure efficacité avec 15 séances de stimulation plutôt que 10 (Pridmore & al, 2000), avec 1200-1600 impulsions par séance plutôt que 800-1000 (Loo & Mitchell, 2005 ; Gershon & al, 2003).

La rTMS cherche sa place au sein de l’éventail des traitements antidépresseurs.

Elle a été comparée aux ECT par rapport auxquels elle présente l’avantage d’être quasiment indolore, de ne pas nécessiter d’anesthésie générale et de ne pas entraîner de troubles cognitifs. Des études ont comparé l’efficacité de la rTMS par rapport à celle des ECT. Les résultats sont contradictoires, certaines études (Grunhaus & al, 2003 ; Janicak & al, 2002) retrouvent des résultats comparables (ou non significativement différents) sur certaines catégories de patients (dépression sans éléments psychotiques) alors que d’autres (Eranti & al, 2007 ; McLoughlin & al, 2007) trouvent une supériorité des ECT sur la rTMS après le traitement mais cette supériorité thérapeutique n’est pas retrouvée au cours du suivi.

Par rapport à un traitement antidépresseur, sa place est difficile à définir dans la mesure où cette thérapeutique est proposée aux patients présentant une résistance médicamenteuse. Cependant il semble que la rTMS en complément à un traitement antidépresseur potentialise l’effet de ces antidépresseurs (Rumi & al, 2006 ; Rossini & al, 2006).

### LIMITES DES ETUDES ACTUELLES

La principale limite des différentes études est leur faible puissance. En effet, elles sont réalisées sur de très petits échantillons limitant la portée des conclusions des analyses statistiques. En outre, ces études constituent un ensemble très hétérogène tant sur le plan des paramètres utilisés que sur les patients recrutés (unipolaire, bipolaire, résistants, avec ou sans traitement). Ainsi, la capacité à démontrer l’efficacité thérapeutique est diminuée avec une telle hétérogénéité.

### ZONE CIBLE : LE CORTEX PREFRONTAL DORSOLATERAL

La plupart des études ciblent le CPFDL gauche (haute fréquence) ou le CPFDL droit (basse fréquence). Son repérage dans le cadre de la rTMS est réalisé selon une méthode anatomique et fonctionnelle décrite par Georges & al, 1995. Elle repose sur le repérage de la zone motrice dont la stimulation entraîne une contraction du court abducteur du pouce. Le CPFDL est positionné statistiquement 5 cm en avant de cette zone dans un plan parasagittal.

Le CPFDL est impliqué dans les tâches de planification, organisation, mémoire de travail et processus attentionnels (Milner & al 1984). Sur un plan cytoarchitectonique, il correspond à l’aire 9/46 de Brodmann. Sur un plan anatomique, il représente la partie moyenne du gyrus frontal moyen (Rajdowsak & al, 1995). Des études d’imagerie fonctionnelle (Mayberg & al, 1999 ; revue de la littérature de Drevets & al, 2000 ; Rogers & al, 2004) ont retrouvé un hypométabolisme de cette région chez les patients déprimés avec un effet de latéralisation droite/gauche. Cet hypométabolisme gauche semble être lié à l’état dépressif dans la mesure où il semble s’amender avec la normalisation de l’humeur. Cependant, l’hypothèse de l’existence d’un hypométabolisme du CPFDL gauche lié à l’état dépressif a été controversée : une méta-analyse sur 23 études d’imagerie fonctionnelle retrouve seulement une diminution diffuse de l’activité cérébrale chez les patients déprimés sans gradient droit/gauche ou antéro-postérieur (Nikolaus & al 2000). Herwig & al, 2003, ont testé également cette hypothèse en stimulant spécifiquement les zones préfrontales de patients déprimés présentant un hypométabolisme mais il n’a pas retrouvé de différence significative lorsque ce groupe est comparé à un autre groupe de patients traités indépendamment du métabolisme cérébral. Les effets antidépresseurs de la rTMS peuvent être liés aux stimulations des nombreux réseaux neuronaux connectés à cette zone dans la mesure où le CPFDL s’intègre dans un large réseau neuronal impliqué dans la régulation de l’humeur (Nahas & al, 2001 ; Kimbrell & al, 2002 Tekin & Cummings, 2002; Li & al, 2004).

## Le problème de la localisation

### LIMITES DE LA METHODE STANDARD

Le repérage du CPFDL tel qu’il a été décrit par Georges & al, 1995 est très imprécis. En effet, les variations anatomiques interindividuelles ne sont pas prises en compte. Cette source d’imprécision a été mise en évidence par Herwig & al, 2001. Le CPFDL est localisé chez 22 patients déprimés selon la méthode standard et comparé à la localisation « réelle » (partie moyenne du gyrus frontal moyen) à l’aide d’un système de neuronavigation. Seuls les CPFDL de 7 patients sont correctement localisés, les autres se trouvant situés trop dorsalement en regard des zones prémotrices. La méthode standard a été améliorée par l’utilisation du système international 10-20 de l’EEG mais la précision reste encore insuffisante (Herwig & al, 2003).

En outre, d’autres sources d’imprécision viennent renforcer l’effet de la variabilité anatomique interindividuelle et en particulier un mode de détermination de la cible opérateur dépendant. Il existe en effet une variabilité dans la localisation du CPFDL à partir de la localisation de la zone motrice du court abducteur du pouce droit en fonction de l’opérateur lorsqu’il a recours à une technique de repérage traditionnelle (C. Nauczyciel, 2007, Mémoire de Master II). Cette localisation dépend d’un grand nombre de variables dont l’intensité de la stimulation et le positionnement de la bobine sur la tête du patient. En effet, l’intensité de stimulation conditionne l’importance de la dépolarisation neuronale induite par cette stimulation. Ainsi, la zone motrice peut-être activée par une stimulation d’intensité suffisante mais pas en regard de cette zone. De même, l’intensité nécessaire à la dépolarisation des neurones du cortex moteur peut varier en fonction de la structure touchée (dépendant du positionnement de la bobine) : gyrus ou sillon (Balselv & al, 2007).

L’intensité de stimulation, en outre, joue un rôle sur la forme du champ magnétique et sur la surface du cortex stimulé (Lontis & al, 2006 ; Thielscher & al, 2004). Ainsi, il existe un risque de dispersion du champ magnétique sur une surface plus importante que celle ciblée. Néanmoins, les conséquences, si elles existent (car cette dispersion du champ au delà de la zone de stimulation ne signifie pas dépolarisation hors de cette zone) n’ont pas été évaluées à notre connaissance.

### CONSEQUENCES ET SOLUTION

Bien que l’importance de la dépolarisation de la zone stimulée dépende aussi de la dispersion du champ magnétique, la localisation du CPFDL telle qu’elle est réalisée avec la méthode standard pose plusieurs questions : l’impact thérapeutique de telles imprécisions, l’évaluation véritable du CPFDL comme zone d’intérêt dans le traitement de la dépression par la rTMS dans la mesure où les études ne sont pas reproductibles. Il est donc nécessaire de travailler avec des outils permettant de s’affranchir des contraintes liées à l’erreur humaine et aux variations anatomiques interindividuelles.

Les études d’Herwig & al utilisent un système de neuronavigation pour localiser le CPFDL en temps-réel sur le patient. Ce système est une adaptation d’outils utilisés en neurochirurgie depuis la fin des années 80. Il permet de réaliser une chirurgie stéréotaxique guidée par l’image en temps réel. Son principe repose sur le repérage des coordonnées 3D (3 dimensions) d’une zone cible (tumeur…) dans un référentiel donné à la fois sur l’imagerie du patient et sur le patient lui-même et de superposer ces deux jeux de coordonnées (voir article de Reinhardt & al, 1999). La localisation précise du CPFDL est donc requise sur l’imagerie IRM du patient.

Ce système est jusqu’alors utilisé dans la TMS pour les études couplées à l’imagerie fonctionnel le (Kleinjung & al, 2006, Herwig & al, 2003 ; Garcia-Toro & al, 2004).

Cette technique est stable, reproductible (Schönfeldt-Lecuona, 2005) et avec une précision supérieure à celle des méthodes standards.

L’utilisation d’un tel outil couplé à la rTMS va permettre de répondre aux questions sur l’efficacité de la rTMS et sur la localisation de manière stable et reproductible de la cible de cette stimulation. Herbsmann & al, 2009 ; Fitzgerald & al, 2009; ont déjà montré l’impact thérapeutique de la localisation du CPFDL. Herbsmann & al, 2009, ont montré l’intérêt d’une stimulation plus latérale et plus antérieure que celle déterminée par la méthode standard. Elle permettait une amélioration thérapeutique supérieure, rejoignant ainsi l’étude de Herwig & al, 2001. Fitzgerald & al, 2009, ont testé l’hypothèse d’une supériorité de l’efficacité thérapeutique de la rTMS couplée à la neuronavigation par rapport à la rTMS seule. Dans cette étude, un neuronavigateur est utilisé pour localiser « off line » et stimuler le CPFDL d’un groupe de 24 patients alors que les 27 autres sont repérés par la méthode standard. Les résultats ont retrouvé une amélioration significative dans le groupe neuronavigué par rapport au groupe standard. Cependant, il existe de nombreux biais : nombreux perdus de vue (29 patients sur 51 ont fini l’étude), système de neuronavigation « off line », localisation sur l’IRM de la région cible. Il est donc nécessaire de confirmer ces résultats en utilisant un outil de neuronavigation permettant d’améliorer la précision et la reproductibilité de la stimulation.

C’est ainsi que le SHU de Psychiatrie de Rennes a collaboré avec l’unité-projet VisAGeS[[1]](#footnote-2) (équipe INRIA[[2]](#footnote-3), unité INSERM U746) pour prototyper un système de neuronavigation. Ce protototype a ensuite été industrialisé, mis en conformité avec la réglementation et est aujourd’hui commercialisé par la société Syneika. La description du système de neuronavigation, ainsi que la procédure proposée, est décrite dans la partie 3.

## Conclusion

La rTMS est une technique de traitement de la dépression cherchant sa place dans l’éventail thérapeutique proposé. Si les études montrent des résultats s’améliorant avec une meilleure connaissance de son mode d’action et des paramètres de stimulation, la méthode de localisation de la zone cible n’a pas évolué et reste très imprécise. Aucune étude n’a, à ce jour, évalué l’impact thérapeutique de cette imprécision et la question de la pertinence du choix de cette zone cible n’a pu être réalisée dans la mesure où les études ne sont pas reproductibles.

Ainsi il est nécessaire de travailler avec un outil palliant les imprécisions de la méthode standard pour réaliser des études fiables et reproductibles évaluant l’impact thérapeutique d’une stimulation précise.

# OBJECTIFS

## Objectif principal

Au vues des données de la littérature, l’objectif de ce projet est d’évaluer par une étude multicentrique randomisée en double aveugle sur deux groupes parallèles, la supériorité thérapeutique de la stimulation localisée du CPFDL gauche par un neuronavigateur par rapport à la stimulation du CPFDL gauche repéré de manière standard dans le traitement des états dépressifs isolés ou récurrents.

Notre hypothèse est qu’une stimulation localisée du CPFDL permet une réponse clinique qualitativement et quantitativement supérieure à une stimulation moins précise.

## Objectifs secondaires

Tester la supériorité du ressenti de l’amélioration thérapeutique chez les patients stimulés avec le système de neuronavigation par rapport aux patients stimulés avec la méthode standard au moyen du Beck Depression Inventory (BDI)

Tester la supériorité de l’amélioration du plan psychomoteur chez les patients stimulés avec le système de neuronavigation par rapport aux patients stimulés avec la méthode standard au moyen de l’Echelle de Dépression et de Ralentissement (EDR)

# SYSTEME DE NEURONAVIGATION

## Principe de fonctionnement

Le système de neuronavigation développé par la société Syneika, elle même exploitant des travaux de l’unité-projet VisAGeS INRIA-INSERM U746, est dérivé des systèmes utilisés en neurochirurgie et adapté aux contraintes de la TMS. Il repose sur un système de suivi 3D optique dans le domaine visible qui permet de suivre dans la scène 3D des objets auxquels sont attachés des marqueurs. Grâce à un tel système, il est possible de fusionner en temps réel les images IRM du patient avec la scène 3D (patient). Cette caméra utilise la vision stéréoscopique pour repérer et suivre dans l’espace quelque soit l’orientation de marqueurs spécifiques. Un marqueur est l’association d’au moins 3 facettes ayant un fort contraste noir/blanc. Il est attaché à un objet spécifique qui peut ainsi être suivi avec une technique de vision par ordinateur (erreur d’oscillation : 0,07 mm).

Pour la rTMS, les marqueurs sont attachés aux objets à suivre : la tête du patient, la bobine et le pointeur pour l’identification des points. Dans cette application, il est nécessaire de visualiser le champ magnétique maximum délivré au centre de la bobine (Cohen & al, 1999) et son orientation autant que la bobine en elle-même. La bobine est donc calibrée en utilisant une mire de calibration fournie par la société Syneika. La précision de la calibration de ce système est de 0,25mm EQM (assurée par le fabriquant).


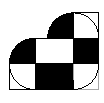


Neuronavigateur Syneika

Exemple de marqueur

La mise en correspondance des images IRM/scène 3D se fait au moyen d’un recalage basé surface : 3 points de repères anatomiques du sujet (face externe de l’œil gauche, nasion, extrémité du nez) sont recalés dans un ordre protocolisé sur les 3 points homologues sur l’IRM déterminés de manière automatique. Puis un échantillonnage de la surface du scalp est réalisé en collectant un grand nombre de points (typiquement quelques centaines) à l’aide du pointeur calibré. Ce nuage de point est ensuite aligné sur la surface du scalp extraite à partir de l’IRM grâce à des algorithmes de type « iterative closest point ». Il a été montré que le recalage basé surface est plus précis que le recalage basé points (West & al, 1999). La précision de ce recalage est comparable à celle des autres systèmes commerciaux (Wirtz & al, 1998)

Par ailleurs, nous avons identifié (C. Nauczyciel, 2007, Mémoire de Master II) que la principale source d’erreur en rTMS neuronaviguée est l’identification du CPFDL sur l’imagerie IRM. Cette localisation requiert une expertise neuroanatomique, et une forte variabilité inter-expert a été identifiée et quantifiée. Nous proposons l’utilisation d’un atlas anatomique (IRM T1 de référence) afin de localiser de manière automatique le CPFDL. Cet atlas est recalé géométriquement sur l’IRM de chaque sujet, en utilisant successivement une transformation rigide puis non-rigide. Ce recalage non-rigide permet de compenser les variations anatomiques inter-individuelles afin de localiser le CPFDL. Ce type de localisation, basé sur un atlas, permet de diminuer fortement la variabilité de stimulation entre chaque centre. Ce type de localisation permet en outre de reporter les points effectivement stimulés dans un référentiel commun (celui de l’atlas et/ou celui de Talairach) afin d’en faire une analyse rétrospective comparative.

Avec un tel système, nous avons donc pu naviguer en temps réel sur le cerveau des patients, stimuler de manière précise et reproductible le CPFDL à chaque séance de TMS et enregistrer les coordonnées du CPFDL réel ou standard (ou de n’importe quelle autre région cérébrale sur laquelle nous voudrions travailler) dans le système de référence de l’IRM.

# SCHEMA DE L’ETUDE

Cet objectif thérapeutique conduit à proposer un essai prospectif multicentrique comparatif en double aveugle sur deux groupes parallèles : repérage de la cible selon la méthode standard versus repérage de la cible par neuronavigateur.

# POPULATION ETUDIEE

## Critères d’inclusion

Sujets volontaires droitiers de plus de 18 ans et de moins de 65 ans :

- Ayant signé un consentement libre et éclairé à la participation à l’étude.
- Répondant au diagnostic d’épisode dépressif majeur (EDM) récurrent ou isolé selon les critères du DSM IV (A.P.A. 1994).
- Ayant un traitement antidépresseur non modifié depuis 3 semaines
- Score à l’échelle MADRS ≥ 21

Le traitement antidépresseur des patients ne doit pas être modifié dans les 3 semaines précédant l’inclusion pour éviter les biais de confusion entre traitement antidépresseur et traitement par rTMS. Les traitements par benzodiazépines doivent être arrêtés dans la mesure où ils peuvent être substitués par d’autres traitements anxiolytiques et qu’ils diminuent l’excitabilité corticale. Les traitements thymorégulateurs, bien qu’ils diminuent également l’excitabilité corticale ne sont pas arrêtés car ils ne peuvent être substitués.

## Critères de non inclusion

- Dépression avec caractéristiques psychotiques
- Diagnostic co-morbide selon l’axe I (DSM IV) de schizophrénie, de dépendance (ou d’abus) à l’alcool et/ou à une autre substance (vie entière)
- Patient répondant aux critères de la dépression résistante définie par le stade V de la classification de Thase & Rush (voir annexe 4)
- Patient hospitalisé sous contrainte ou sous mesure de protection juridique (tutelle, curatelle)
- Patient présentant un risque suicidaire élevé (item 10 MADRS > 3) en l’absence d’une hospitalisation
- Contre-indication à la pratique de l’IRM ou la rTMS : antécédents personnels de crise comitiale, de pathologies neurologiques ou neurochirurgicales, matériel prothétique métallique ou corps étrangers (pacemaker, matériel ferromagnétique intra oculaire)
- Patient de plus de 65 ans en raison du risque d’atrophie corticale.
- Grossesse.

# RANDOMISATION

## Constitution de la liste de randomisation

La randomisation est stratifiée selon le centre, les listes ayant été établies avec un logiciel spécifique par les responsables du data management.

### Modalités pratiques de la randomisation

Après vérification des critères d’inclusion et de non inclusion, l’investigateur procède à la randomisation du malade.

La procédure de randomisation est la suivante :

- L’investigateur se connecte sur le cahier d’observation électronique de l’étude, complète les critères d’inclusion et de non-inclusion et valide l’enregistrement du patient.

- Le logiciel du cahier d’observation (logiciel Capture système, Clinsight, Cenon (33), France , Cf. paragraphe 15.7) intègre les données saisies par l’investigateur et attribue au malade le premier numéro disponible. L’investigateur réalisant les rTMS est informé en ligne du bras dans lequel le patient est inclus (sans en informer l’investigateur cotateur pour garantir le double aveugle).

**Le patient est randomisé lors de la visite d’inclusion.**

### Modalités de levée du code de randomisation

Le code de randomisation ne peut être levé que lors de la survenue d’un évènement indésirable grave dont la prise en charge nécessite de façon absolue la connaissance du traitement par l’investigateur. Avant toute levée du code de randomisation, l’investigateur devra contacter, si possible, l’investigateur principal ou le méthodologiste de l’étude.

En cas de décision de levée du code pour un participant, l’investigateur devra appeler le pharmacien qui possède la randomisation de l’essai et qui lui transmettra le nom du traitement administré.

Dès que la levée du code de randomisation est effectuée, l’investigateur transmet par fax au méthodologiste de l’étude les éléments suivants :

1. deux premières lettres du nom, première lettre du prénom et date de naissance du malade,
2. numéro de randomisation,
3. date et heure de la levée du code de randomisation,
4. raison de la levée du code de randomisation,
5. nom du médecin ayant effectué la levée du code de randomisation.

En cas de levée du code de randomisation, le suivi du malade sera néanmoins effectué, si possible, jusqu’à la fin de l’essai selon les procédures décrites dans le protocole.

# DEROULEMENT DE L’ETUDE

Le traitement de fond habituel (antidépresseur et/ou thymorégulateur) du patient ne sera pas modifié pendant les 3 semaines précédant l’étude et jusqu’à l’évaluation finale excepté en cas d’urgence thérapeutique pour laquelle un changement pourra être effectué dans l’intérêt du patient. Dans ce cas, les patients seront suivis et analysés en respect du principe de l’intention de traiter.

La prescription et ou les modifications de prescription concernant d’autres traitements à visée symptomatique (anxiolytiques et hypnotiques) est autorisée. Les antihistaminiques seront à privilégier. En cas de nécessité, les autres traitements anxiolytiques ou hypnotiques pourront être prescrits selon les recommandations en vigueur. Toute modification du traitement sera reportée dans le cahier d’observation.

## Phase d’éligibilité

Les patients susceptibles d’être inclus sont sélectionnés par les médecins investigateurs au cours de consultations en ambulatoire ou au cours d’une hospitalisation.

## Phase d’inclusion et suivi thérapeutique des patients

Avant l’inclusion, les patients passent un entretien médical permettant de vérifier les critères d’inclusion et d’exclure les patients à risques de crise comitiale et les patients ayant des corps ferromagnétiques intra oculaires. Un test de grossesse est réalisé pour les femmes en âge de procréer.

Les patients ainsi sélectionnés sont randomisés lorsqu’ils ont donné leur consentement libre et éclairé après avoir reçu une information exhaustive sur le protocole.

Quel que soit le bras dans lequel ont été randomisés les patients, tous doivent avoir passé une IRM 3D nécessaire à la neuronavigation.

- Si un patient a déjà bénéficié au cours de son suivi habituel d'une IRM 3D alors celle-ci sera utilisée sans en refaire ;

- Si un patient n'a jamais bénéficié d'une IRM 3D, alors celle-ci devra être réalisée dans les 15 jours suivant la randomisation.

Les patients, après avoir été randomisés, sont affectés à un des traitements suivants :

- rTMS à haute fréquence (20Hz) appliquée au niveau du CPFDL gauche repéré par neuronavigation

- rTMS à haute fréquence (20Hz) appliquée au niveau du CPFDL gauche repéré par la méthode standard dite « des 5 cm »

La première séance de TMS doit être effectuée au plus tard 15 jours après la randomisation. Elle définit le J0 de l’étude

Le traitement est délivré par un clinicien différent de l’investigateur-cotateur, informé par le CIC du bras de randomisation.

Les échelles MADRS, BDI et ERD sont réalisées par l’investigateur-cotateur aveugle au bras de traitement, avant la première séance de TMS.

Après les 10 séances de traitement par rTMS, les patients entrent dans une phase de suivi au cours duquel ils sont réévalués 2 fois à l’aide de ces même échelles: après la dernière séance (J14) et 1 mois après la dernière séance (J44).

Le traitement antidépresseur, stable depuis au moins 3 semaines ne sera modifié qu’en cas d‘urgence thérapeutique. Toute modification sera reportée dans le cahier d’observation.

En cas de risque suicidaire majeur (item 10 MADRS> 3), les patients seront hospitalisés conformément aux bonnes pratiques cliniques.

**Schéma du protocole :**

INCLUSION

10 SEANCES TMS

SUIVI DES PATIENTS

Absence de CI

IRM

Neuronavigation ou méthode des 5cm

Echelles : MADRS, BDI, EDR

J0

J14

J44

Une souplesse de trois jours avant ou trois jours après les visites J14 et J44 est tolérée.

A l’issue du protocole les patients bénéficient d’une prise en charge de leur pathologie dépressive par leur psychiatre habituel. Les patients répondeurs à la rTMS pourront bénéficier de nouvelles séances de rTMS en cas de rechute ou de récurrence des troubles dépressifs, après évaluation conjointe du psychiatre traitant et du psychiatre responsable des soins par rTMS. Les patients non répondeurs à la rTMS seront pris en charge selon les méthodes thérapeutiques usuelles de la dépression. En particulier un ajustement de leur traitement antidépresseur leur sera proposé, avec prescription d’un antidépresseur de classe différente. Le suivi du patient sera confié à son psychiatre traitant.

## Protocole thérapeutique de la rTMS et neuronavigation

### La Stimulation magnétique transcrânienne

Les rTMS sont effectuées à l’aide de stimulateur de type Mag Pro (société Magventure, Danemark) ou équivalent, aux normes CE. Ce stimulateur est adapté à une utilisation dans un but thérapeutique avec une capacité à délivrer des stimulations à haute fréquence selon un programme informatisé. Il sert également à déterminer le seuil moteur. Les stimulations sont délivrées avec une bobine en huit permettant de stimuler une région focale du cortex cérébral.

Les séances de rTMS sont réalisées quotidiennement à raison de 5 par semaine, 2 semaines consécutives.

Les critères de stimulations sont adaptés aux connaissances actuelles de la littérature conformément à ceux utilisés lors des études récemment publiées et dans le respect des recommandations de sécurité. Nous nous basons en particulier sur les paramètres de stimulations utilisés par Machii & al, 2006 lors d’une étude sur la sécurité et la tolérance chez 249 sujets sains et 249 sujets déprimés ainsi que sur l’étude de Chen & al, 1997.

**Paramètres de stimulation** :

- Fréquence : 20Hz
- Intensité : 110% du seuil moteur
- Nombre de séances : 10
- Nombre de train par séance : 80
- Durée d’un train : 2 secondes
- Durée de l’intervalle inter-train : 10secondes
- Nombre total d’impulsions par séance : 3200

L’intensité de stimulation sera déterminée lors de la première séance pour réaliser une mesure du seuil moteur des sujets. Il correspond à l’intensité minimale permettant d’obtenir dans 50% des cas au moins une contraction de l’abducteur du pouce droit par stimulation de la zone motrice corticale correspondante.

Quelque soit le bras dans lequel sont randomisés les patients, ceux ci bénéficient d’une double détermination de l’intensité de stimulation : détermination selon la méthode précédemment décrite et vérification de la localisation correcte du cortex moteur par neuronavigation. L’intensité minimale déterminée servira d’intensité de référence.

### Localisation du cortex préfrontal dorsolatéral (CPFDL G)

Tous les centres sont équipés du système de neuronavigation Syneika One.

Les patients reçoivent une double détermination du CPFDL G (standard et par neuronavigation) afin de réaliser les mesures de distance entre ces 2 points.

La localisation dite standard du CPFDL G est réalisée selon la méthode décrite par Georges & al, 1995 : le CPFDL G est situé 5cm en avant de la zone précédemment déterminée sur le cortex moteur dans un plan parasagittal.

La localisation par neuronavigation se fait à partir de la détermination sur l’IRM du patient du CPFDL G défini comme la partie moyenne du gyrus frontal moyen. Celui-ci est effectué automatiquement par la fonction « localisation automatique » du neuronavigateur pour éviter un effet centre. Cette méthode échoue à localiser le CPFDL G dans environ 10 % des cas : la localisation se fera alors par un investigateur expérimenté dans chaque centre à partir de l’IRM du patient.

Les 2 points déterminés sont enregistrés dans le logiciel de neuronavigation.

En fonction du bras dans lequel les patients ont été randomisés, la stimulation est réalisée en regard de l’une ou l’autre des 2 cibles déterminées.

**Condition d’aveugle pour le patient :**

- le système de neuronavigation est systématiquement utilisé pour positionner la bobine sur le scalp du patient soit au dessus de la cible « réelle », soit au dessus de la cible « standard »
- les patients ne savent pas quelle cible est stimulée

# PARAMETRES ETUDIES

## Description des outils

### Outils diagnostics

Le diagnostic de dépression et l’exclusion des autres co-morbidités telles que la schizophrénie seront réalisés à partir d’un entretien clinique et de l’entretien structuré avec le MINI 5.0

Le MINI 5.0 permet de conforter des diagnostics psychiatriques en se basant sur le Manuel diagnostique et statistique des troubles mentaux (DSM IV)

Il se base sur un questionnaire vie entière passé au cours d’un entretien standardisé explorant les principaux troubles psychiatriques de l’axe 1 du DSM IV. Cet entretien sera complété d’un entretien clinique non standardisé afin d’éliminer d’éventuels faux positifs.

Le MINI 5.0 est structuré en plusieurs chapitres explorant chacun une catégorie diagnostique psychiatrique du DSM IV. Au début de chaque chapitre, plusieurs questions spécifiques du trouble exploré permettent de filtrer les réponses et de n’explorer que les chapitres pertinents. A la fin de chaque chapitre, des boîtes diagnostiques permettent au médecin d’indiquer si les critères diagnostiques sont atteints.

### Evaluations cliniques

L’intensité de la dépression est évaluée à l’aide des scores à l’échelle Montgomery & Asberg Depressive Rating Scale (MADRS), de l’échelle de ralentissement de Widlocher (ERD) et de l’autoquestionnaire de Beck de 13 items (révisé en 1996)

**Echelle de dépression MADRS**

La MADRS est une échelle relativement simple, courte, de passation assez facile. Elle est sensible au changement, différencie bien les différents degrés de gravité de la dépression et constitue donc un bon indice global. Elle est à l'heure actuelle très utilisée dans les essais de psychotropes et lorsque l'on veut avoir un témoin de l'intensité dépressive comme variable de contrôle. Elle présente également une bonne sensibilité aux changements thymiques et une bonne aptitude à distinguer différents degrés de dépression (Kearnns & al, 1982).

Elle est constituée de 10 items explorant différents aspects de la dépression en mettant l’accent sur l’aspect psychologique plutôt que somatique (Galinowsky & al, 1995). Chaque item a 6 degrés de gravité. La note seuil pour la dépression a été fixée à 15. La note minimum pour l’inclusion dans un essai thérapeutique est de 20-21.

**Echelle de ralentissement dépressif de Widlocher (ERD)**

L’échelle de ralentissement de Widlocher est un instrument mesurant le ralentissement psychomoteur, noyau commun aux différents états dépressifs. Il complète l’évaluation de la dépression réalisée avec la MADRS dans la mesure où il s’intéresse aux aspects somatiques.

Elle est constituée de 14 items décrivant le comportement moteur, verbal, idéique, hédonique et cognitif des patients déprimés. Chaque item est pondéré de 0 à 4.

Elle présente une bonne sensibilité au changement.

**Auto questionnaire de Beck (BDI)**

L’auto questionnaire de Beck est un des inventaires les plus utilisé en recherche et en clinique pour estimer l’intensité des sentiments dépressifs sur un plan subjectif (Gallagher, Nies & Thompson, 1982). Si la version originale de 1961 est inappropriée actuellement dans la mesure où elle présentait des items sur la dépression telle qu’elle était conceptualisée à l’époque, la version révisée de 1996 répond mieux aux critères du DSM IV.

Elle comporte 13 items classés de 0 à 3 sur les symptômes et les manifestations comportementales spécifiques de la dépression. Elle est donc une échelle de passation rapide, facile à remplir et de bonne sensibilité.

## Paramètres évalués

### A l’inclusion

- Données socio démographiques
- Antécédents psychiatriques
- Durée et traitement de l’épisode actuel
- MINI, MADRS.

### Paramètres évalués durant les séances de TMS

Le type de localisation du CPFDL G (automatique ou manuelle) sera notée.

La valeur du seuil moteur sera recueillie lors de la première séance de chaque semaine. Les coordonnées de la zone stimulée et l’intensité de la stimulation seront recueillies au cours des séances de TMS.

Les coordonnées seront exprimées en millimètres dans le repère du patient, sous la forme suivante :

- x : droite -> gauche

- y : antérieur -> postérieur

- z : bas -> haut

L'origine du repère est donc dans le coin droit antérieur bas de l'image."

### Paramètres évalués lors des visites de suivi

Les évaluations cliniques MADRS, BDI et ERD sont réalisées par un clinicien aveugle aux conditions de stimulation des patients. Elles sont réalisées à la fin du traitement par rTMS et un mois après la fin du traitement.

A chaque séance, la tolérance et les effets secondaires sont notés sur le cahier d’observation.

# CRITERES DE JUGEMENT

## Critère de jugement principal

Le critère de jugement principal est l’obtention d’une réponse clinique définie par une diminution de la moitié du score de la MADRS à J44, à l’issue du traitement par TMS et de la période de suivi. »

## Critères de jugement secondaire

Un des critères de jugement secondaire est le pourcentage de patients présentant une rémission clinique à J14 et J44.

La rémission clinique est définie par un score à la MADRS inférieur ou égal à 8.

Le pourcentage de patients répondeurs à J14 est également un critère secondaire

Le ressenti de l’amélioration clinique est également évalué via la variation de l’échelle BDI après le traitement à J14 et au cours du suivi (J44) dans chaque groupe

Le ralentissement psychomoteur est évalué via la variation de l’échelle ERD après le traitement et au cours du suivi dans chaque groupe.

# NOMBRE DE SUJETS NECESSAIRE

La question posée est en formulation bilatérale.

Le taux attendu de patients améliorés (MADRS diminuée de 50%) dans le bras « stimulation standard » est de 35%. Le bénéfice attendu avec la neuronavigation doit permettre d’augmenter ce taux à 70% Si l’on veut se donner les moyens statistiques de détecter une augmentation absolue de 35% dans le bras « neuronavigation » il faut inclure 60 sujets par bras pour garantir une puissance de 95% dans un test réalisé avec un risque d’erreur α = 5%.

Le nombre total de sujets nécessaire est donc de 120.

# ANALYSE STATISTIQUE

L’analyse statistique principale porte sur tous les patients randomisés et évalués (analyse en intention de traiter). Elle est réalisée avec le logiciel SAS (SAS Institute, Cary, NC, USA) dans l’Unité de Pharmacologie Clinique du CIC de Rennes. Elle est effectuée à partir des données recueillies dans le cahier d’observation de chaque sujet. Le monitoring et le data management sont aussi assurés par l’Unité de Pharmacologie Clinique du CIC de Rennes.

Les éventuels patients devant recevoir en cas d'urgence une modification de leur traitement habituel seront considérés comme échecs sur le critère de jugement principal et les critères secondaires qualitatifs c'est-à-dire comme non répondeurs et n'étant pas en rémission. Ils seront pris en compte dans l'analyse, dans le bras où ils ont été randomisés, en respect du principe de l'analyse en intention de traiter.

Il est prévu de réaliser une analyse de sensibilité sur la population en per protocole. Celle-ci se définit comme la population ayant reçu le traitement à l’étude conformément au protocole et ayant bénéficié d’une localisation automatique pour les patients neuronavigués.

## Analyse descriptive

Une première analyse descriptive globale et par groupe est réalisée. Elle comporte des estimations ponctuelles, nombres et pourcentages pour les variables qualitatives, moyennes, écart-types, médianes et intervalles interquartiles pour les variables quantitatives. La normalité de la distribution des variables quantitatives est vérifiée.

## Comparaison des groupes en fonction du traitement reçu

### Comparabilité des groupes à l’inclusion

Test du Chi² ou test exact de Fisher si nécessaire pour les variables qualitatives

Test t de Student ou test de Wilcoxon si nécessaire pour les variables quantitatives.

### Analyse sur le critère principal

Test du chi2  et analyses ajustées sur les principales variables pronostiques ou déséquilibrées à l’inclusion à l’aide d’un modèle de régression logistique.

### Analyses sur les autres critères

Test du Chi² ou test exact de Fisher si nécessaire pour les variables qualitatives

Test t de Student ou test de Wilcoxon si nécessaire pour les variables quantitatives.

## Analyse des évènements indésirables

Les éventuels évènements indésirables sont codés selon la classification MedDRA et font l’objet d’une analyse descriptive.

# GESTION DES EVENEMENTS INDESIRABLES

## Définitions

**Evénement indésirable (EvI)** : toute manifestation nocive survenant chez une personne qui se prête à une recherche biomédicale que cette manifestation soit liée ou non à la recherche ou au produit sur lequel porte cette recherche.

**Evénement indésirable grave (EvIG)** : la gravité est définie par l’une des constatations suivantes :

- Décès
- Mise en jeu du pronostic vital
- Incapacité ou handicap important ou durable
- Hospitalisation
- Prolongation d’hospitalisation
- Malformation/anomalie congénitale
- Evènement potentiellement grave (évènement clinique indésirable ou résultat de laboratoire à caractère grave ou considéré comme tel par l’investigateur)

**Effet indésirable (EI)** : toute réaction nocive et non désirée à la recherche.

**Effet indésirable grave (EIG)**: évènement indésirable grave imputable à la recherche. **Effet indésirable inattendu** : effet indésirable dont la nature, la sévérité, l’intensité ou l’évolution ne concorde pas avec les informations figurant dans le protocole.

## Rôle de l’investigateur

### Déclaration des évènements indésirables graves

- **Période de notification au promoteur**

L’investigateur a la responsabilité de noter et de rapporter tous les évènements indésirables graves survenant pendant l’ensemble de l’étude : entre l’inclusion et la fin du suivi du patient.

Par ailleurs, quel que soit le délai de survenue après la fin de l’étude, tout évènement indésirable grave susceptible d’être dû à la recherche doit être déclaré au promoteur dès lors qu’aucune autre cause que la recherche ne peut raisonnablement lui être attribuée.

- **Délai de notification au promoteur**

Tout EvIG doit impérativement être déclaré dans les **24 heures** de sa survenue (ou dès que le médecin investigateur en a connaissance) au promoteur de l’étude, en faxant le formulaire de déclaration des EvIG provenant du cahier d’observation.

- **Spécificités du protocole**

Sont considérés comme graves plus spécifiquement pour ce protocole, les évènements suivants :

Crise convulsive tonico-clonique généralisée,

Céphalées résistantes aux antalgiques de niveau 1

- **Les hospitalisations** ne seront pas considérées comme des évènements indésirables graves dans les cas suivants :

Traitement de routine ou surveillance de la pathologie étudiée non associé à une détérioration de l’état du patient,

Traitement programmé pour une affection préexistante à l’inclusion et ne présentant pas de signes d’aggravation,

- **Informations à transmettre au promoteur**

Chaque évènement indésirable grave sera décrit sur la fiche dédiée à cet effet (« Déclaration Initiale d’Événement Indésirable » ou « Déclaration de Suivi d’Événement Indésirable ») en essayant d’être le plus exhaustif possible.

Chaque évènement indésirable grave sera suivi jusqu’à sa stabilisation ou à un niveau jugé acceptable par l’investigateur ou retour à l’état antérieur, même si la patient est sorti d’essai..

### Déclaration des événements indésirables non graves

Tous les autres EvI seront rapportés sur le formulaire « évènement indésirable » du cahier d’observation en précisant la date de survenue, la description, l’intensité, la durée, le mode de résolution, l’étiologie, l’imputabilité et les décisions prises.

### Conduite à tenir devant un effet indésirable

**Si un EI survient, l’investigateur pourra** :

- soit poursuivre la procédure expérimentale
- Soit arrêter temporairement ou définitivement la procédure expérimentale

En outre, la surveillance sera renforcée et un traitement symptomatique pourra être ajouté.

## Rôle du promoteur

### Analyse des événements indésirables graves

Afin de conclure sur la nécessité d’une déclaration aux Autorités Compétentes (AC) et au Comité de Protection des Personnes (CPP), l’analyse des EvIG sera réalisée selon la procédure de gestion des EvIG en vigueur au CIC de Rennes. Cette procédure décrit le fonctionnement d’un « comité EvIG » associant des représentants du promoteur, du CIC, et du Centre Régional de Pharmacovigilance (CRPV). Ce comité évalue l’imputabilité et le caractère attendu ou inattendu des EvIG.

Seuls les EvIG dont l’imputabilité sera « lié à la procédure expérimentale » et dont le caractère sera « inattendu » seront transmis aux AC et au CPP dans un délai de 7 jours en cas de décès ou de mise en jeu du pronostic vital, ou de 15 jours dans les autres cas.

### Cotation de l’imputabilité

- Conformément aux recommandations ICH sur la gestion des évènements indésirables dans les essais cliniques - ICH E2B(R3), version du 12 mai 2005 - une évaluation de l’imputabilité est réalisée pour tout EvIG déclaré en utilisant 2 conclusions possibles : « reasonnable possibility  » et «reasonnable possibility» (termes Eudravigilance).

# PERDUS DE VUE ET SORTIE D’ESSAI

L’investigateur est responsable de la surveillance des malades inclus dans son centre. Il est donc nécessaire de mettre en œuvre de façon rigoureuse les procédures de surveillance prévues par le protocole (respect des dates de consultation, bonne coordination des rendez vous pour la réalisation des examens complémentaires...), **et ceci même si le traitement de l’étude a été arrêté de façon temporaire ou définitive.**

## Procédure de gestion des perdus de vue

Dans l’hypothèse où le patient ne se présenterait pas à une consultation, il est indispensable que l’investigateur prévienne immédiatement l’ARC du CIC de Rennes en charge du protocole et mette en œuvre tous les moyens dont il dispose pour obtenir les raisons de l’absence du malade et les informations requises par le protocole.

## Procédure de gestion des sorties d’essai

Les volontaires peuvent être exclus de l’étude dans les cas suivants :

- retrait du consentement du sujet lui-même,
- volonté de l’investigateur,
- décès du patient.

Les motifs de sortie d’étude seront clairement indiqués dans le cahier d’observation.

# SURVEILLANCE DE L’ESSAI

## Centre de Coordination de l’étude

Le centre de coordination de l’étude est le centre de Rennes. L’investigateur principal/coordonnateur de l’étude est le Pr D. Drapier et le Pr B.Millet est investigateur coordonnateur.

## Comité de pilotage

Le comité de pilotage est composé des personnes suivantes : Pr Millet, Pr Drapier, Mr Hellier, Dr Reymann, Dr Nauczyciel.

Il a pour mission de régler les problèmes d’ordre scientifique, méthodologique et éthique posés par l’essai et de surveiller son bon déroulement. Il se réunit au moins 2 fois par an, mais des réunions extraordinaires peuvent avoir lieu à la demande de l’investigateur principal ou du méthodologiste si des évènements indésirables graves ou des résultats pouvant remettre en cause l’existence du protocole surviennent. Il représente l’instance de décision de l’essai et assure l’information régulière de l’ensemble des participants.

## Comité de Validation des Données

Le Comité de Validation des Données a pour mission de s’assurer que le malade inclus répond bien aux critères d’inclusion sans présenter de critère de non inclusion et de valider le critère de jugement principal. Il travaille en aveugle du traitement. Il est constitué à l’initiation de l’étude et comprend 5 membres dont l’investigateur principal, le méthodologiste, et des personnes choisies sur leurs compétences dans les champs d’investigation de l’essai.

# ASPECTS LOGISTIQUES, LEGAUX ET GENERAUX

## Calendrier prévisionnel

Date de début : janvier 2013

Période de recrutement : 4 ans

Durée de suivi : 59 jours

Durée totale de l’étude : 52 mois

Date de fin d'étude : avril 2017

## Faisabilité de l’étude

La technique de traitement de la dépression par rTMS est innovante et relativement récente puisqu’elle a été réellement développée depuis une dizaine d’années en France et reconnue par la FDA pour la dépression en 2008. Sa mise en œuvre nécessite un investissement initial pour l’acquisition de l’appareil et une formation adéquate. D’autre part, comme toute technique innovante, une dimension de recherche importante lui est associée. Ainsi, dans la pratique quotidienne, les appareils de rTMS sont localisés dans les CHU qui ont une politique de promotion et de recherche de nouveaux outils thérapeutiques. Ces raisons font que peu d’appareils à rTMS sont disponibles par région, rapportés au nombre de patients susceptibles d’en bénéficier. Dans ces conditions le recrutement des patients est relativement aisé puisqu’il intéresse les patients relevant du CHU concerné mais aussi tous les patients dépendants des structures hospitalières avoisinantes. De plus cet outil n’est pas encore développé dans une pratique de soins libérale, ce qui contribue à la qualité du recrutement des patients.

## Page de signature du protocole

Un document, indiquant que la recherche sera conduite conformément au protocole, aux bonnes pratiques cliniques et aux dispositions législatives et règlementaires en vigueur, est signé par les investigateurs avant le début de l’étude et archivé.

## Soumission du protocole au CCP et déclaration à l’autorité compétente

Le promoteur de l’étude, le CHU de Rennes soumettra ce protocole au CPP de Brest et à l’autorité compétente en accord avec la loi n° 2004-806 du 9 août 2004 relative à la politique de santé publique.

## Amendement au protocole

Tout amendement au protocole de l’étude sera notifié au CPP de Brest et à l’autorité compétente s’il entraîne des modifications substantielles, c’est-à-dire si les modifications ont un impact significatif sur tout aspect de la recherche, notamment sur la protection des personnes, sur les conditions de validité de la recherche, le cas échéant sur la qualité et la sécurité des produits expérimentés, sur le déroulement de la recherche ou sur les modalités de conduite de celle-ci.

## Notice d’information au patient et consentement

Les sujets ne peuvent participer à cette étude que s’ils donnent leur consentement libre, éclairé et par écrit.

Les éléments mentionnés sur la notice d’information portent sur :

- le nom du promoteur et l’assurance souscrite par le promoteur dans le cadre du projet,
- l’objectif, le déroulement de la recherche,
- les alternatives aux produits étudiés ou à l’étude proposée,
- les bénéfices attendus, contraintes et risques prévisibles,
- le droit pour les personnes sollicitées de refuser de participer à la recherche ou de retirer leur consentement à tout moment sans encourir aucune responsabilité ni atteinte à la qualité des soins,
- la possibilité d’obtenir toute information complémentaire ainsi que les résultats globaux à l’issue de la recherche,
- la confidentialité des données recueillies,
- l’avis du CPP.

L’ensemble de ces informations est résumé sur une notice d’information et associé à un formulaire de consentement de participation daté et paginé qui est remis à chaque patient.

Une copie de ce document sera remise à la personne participant à l’étude, l’investigateur principal gardera le deuxième exemplaire dans ses archives pendant un minimum de 15 ans.

## Transcription des données et archivage

Toutes les informations requises par le protocole doivent être consignées dans les cahiers d’observation et une explication doit être apportée pour chaque donnée manquante. Les données devront être recueillies au fur et à mesure qu'elles sont obtenues et enregistrées de façon explicite dans ces cahiers.

Un cahier d’observation électronique sera mis en place et la saisie se fera dans les centres par l’intermédiaire d’une interface WEB (logiciel Capture système, Clinsight, Cenon (33), France). Il requiert uniquement une connexion Internet et un navigateur. Un document d’aide pour l’utilisation de cet outil sera fourni aux investigateurs. L’interface entre l’ARC et l’investigateur sera ainsi favorisée, permettant le recueil, le contrôle des données à distance. Les tests de contrôle de cohérence des données seront intégrés au format électronique. Une fonction d’audit trail est intégrée au cahier électronique permettant ainsi de suivre toute modification de données de l’étude. Cette fonction permet également d’identifier clairement la personne ayant fait la modification ainsi que la date. Une justification peut éventuellement être intégrée en commentaire.

Une impression papier sera demandée en fin d'étude, authentifiée (datée et signée) par l'investigateur. Une copie du document authentifié à destination du promoteur devra être archivée par l’investigateur.

## Saisie des données

La saisie des données sera réalisée sur un support électronique via un navigateur internet.

L’analyse des données sera réalisée par l’unité de biométrie du centre d’investigation clinique de Rennes.

L’utilisation de ce fichier informatique est réalisée conformément à la méthodologie de référence MR001 éditée par la CNIL.

## Responsabilité

Le CHU de Rennes est le promoteur de cette étude. En accord avec la loi n° 2004-806 du 9 août 2004 relative à la politique de santé publique, il a pris une assurance couvrant sa responsabilité civile pour les dommages potentiels qui pourraient survenir aux sujets participant à cette étude.

## Rapports fournis en cours et en fin d’étude

Une fois par an pendant toute la durée de l’essai, le promoteur transmet à l’autorité compétente un rapport de sécurité conformément à la loi n° 2004-806 du 9 août 2004 relative à la politique de santé publique. Un résumé de fin d’étude est également transmis à l’autorité compétente

Le processus de gestion des données prévoit la rédaction d’un rapport de qualité des données et d’un rapport statistique en fin d’étude.

## REGLES DE PUBLICATION

Les règles de publication sont les suivantes :

- signature par l’investigateur principal, le méthodologiste et les co-investigateurs au prorata du nombre de malades inclus et dans la limite du nombre d’auteurs acceptés par la revue, puis par ceux qui viendraient apporter une contribution significative au cours de son déroulement,
- mention de l’origine du financement,
- mention de l’origine des produits étudiés.

En cas d’études annexes, les résultats de celles-ci ne pourront être publiés qu’avec l’accord de l’investigateur principal et du méthodologiste, et uniquement après publication de l’étude principale qui devra être citée.

# LE BUDGET

Le budget total de cette étude est en annexe

# References bibliographiques

**Anderson B, Mishory A, Nahas Z, Borckardt JJ, Yamanaka K, Rastogi K & al** ; Tolerability and safety of high daily of repetitive transcranial magnetic stimulation in healthy young men ; 2006 ; J ECT 22 : 49-53

**Avery DA, Holzheimer PE, Fawaz W & al** ; A controlled study of repetitive transcranial magnetic stimulation in medication-resistant major depression ; 2006; Biol Psychiatry 59: 187-194

**Balselv D, Braet W, McAllister C, Miall RC**; Inter-individual variability in optimal current direction for transcranial magnetic stimulation of the motor cortex; 2007; J of Neuroscience Methods 162: 309-313

**Barker AT, Jalinous R, Freeston IL**; Non invasive stimulation of the human motor cortex ;1985 ; Lancet 1 : 1106-1107

**Beck AT;** Beck Depression Inventory; 1971 révisé 1996

**Chen R, Classen J, Gerloff C & al**; Depression of motor cortex excitability by low-frequency transcranial magnetic stimulation 1997 ; Neurology 48 : 1398-1403

**Chen R, Gerloff C, Classen J, Wassermann EM, Hallett M, Cohen LG;** Safety of different inter-train intervals for repetitive transcranial magnetic stimulation and recommendations for safe range of stimulation parameters; 1997; Electroencephalog Clin Neurophysiology 105: 415-421

**Cohen LG, Roth BJ, Nilson J, Dang N, Panizza M, Bandinelli S**; Effects of coils design on delivery of focal magnetic stimulation. Technical considerations; Electroencephalog Clin Neurophysiology; 1999; 75 : 350-357

**Couturier J;** Efficacity of rapide-rate repetitive transcranial magnetic stimulation in the treatment of depression: a systematic review and meta-analyse; J Psychiatry Neurosci; 2005; 30:83-90

**Drevets WC;** Neuroimaging Studies of Mood Disorders*;* Biol Psychiatry; 2000; 48:813-829

**Eranti S, Mogg A, Pluck G & al**; A randomized, controlled trial with 6-month follow-up of repetitive transcranial magnetic stimulation and electroconvulsive therapy for severe depression ; 2007 ; Am J Psychiatry 164 : 73-81

**Eschweiler GW, Wegener C, Schlotter W & al**; Left prefrontal activation predicts therapeutic effects of repetitive transcranial magnetic stimulation in major depression; 2000, Psychiatry Res Neuroimaging 99: 161-172

**Fava M;** diagnosis and definition of treatment resistant depression; 2003; Biol Psychiatry 53: 649-659

**Fitzgerald PB, Brown TL, Marston NA, Daskalakis ZJ, De Castella A, Kulkarni J** ; Transcranial magnetic stimulation in the treatment of depression : a double-blind, placebo-controlled trial ; 2003 ; Arch Gen Psychiatry 60 : 1002-1008

**Fitzgerald PB, Hoy K, McQueen S, Maller JJ, Herring S, Segrave R & al;** A randomized trial of rTMS targeted with MRI based neuro-navigation in treatment-resistant depression; 2009; Neuropsychopharmacology 34: 1255-1262.

**Galinowski A, Lehert P**; Structural validity of MADRS during antidepressant treatment; 1995; Int Clin Psychopharmacol 10 : 157-161

**Gallagher D, Nies G, Thompson LW**; Reliability of the Beck Depression Inventory with older adults; 1982 ; J Consult Clin Psychol 50 : 152-153

**Geddes & UK ECT Review Group;** Efficacy and safety of electroconvulsive therapy in depressive disorders: a systematic review and meta-analysis; 2003; Lancet 361: 799-808

**Gershon AA, Dannon PN, Grunhaus L**; Transcranial magnetic stimulation in the treatment of depression; 2003 ; Am J Psychiatry 160 : 835-845

**George MS, Wassermann EM, Kimbrell TA & al** ; Mood improvement following daily left prefrontal repetitive transcranial magnetic stimulation in patients with depression: a placebo-controlled crossover trial ; 1997 ; Am J Psychiatry 154 : 1752-1756

**George MS, Wassermann EM, Willams WA, Callahan A, Ketter TA, Basser & al**; Daily repetitive trancranial magnetic stimulation improves in mood depression ; 1995 ; NeuroReport 14 : 1853-1856

**George MS, Wasserman EM, Williams WA, Steppel J, Pascual-Leone A, Basser P**; Changes in mood and hormone levels after rTMS of the prefrontal cortex; 1996; J Neuropsychiatry Clin Neurosci 8: 172-180

**Gross M, Nakamura L, Pascual-Leone A, Fregni F**; Has repetitive transcranial magnetic stimulation treatment for depression improved? A systematic review and meta-analysis comparing the recent vs the earlier rTMS studies; 2007; Acta Psychiatria Scand 116: 165-173

**Grunhaus L, Schreiber S, Dolberg OT, Polak D, Dannon PA** ; A randomized controlled comparison of electroconvulsive therapy and repetitive transcranial magnetic stimulation in severe and resistant nonpsychotic major depression ; 2003 ; Biol Psychiatry 53 : 324-331

**Herbsman T, Avery D, Ramsey D, Holtzheimer P, Wadjik C, Hardaway F,& al**; More lateral and anterior prefrontal coil

location is associated with better repetitive transcranial magnetic stimulation antidepressant response; 2009 ; Biol Psychatry 66 : 509-515.

**Herrmann LL, Ebmeier KP**; Factors modifying the efficacy of transcranial magnetic stimulation in the treatment of depression: a review; 2006; J Clin Psychiatry 67: 1870-18776

**Herwig U, Lampe Y, Juengling FD & al**; Add-o, for treatment of depression: a pilot study using stereotaxic coil-navigation according to PET data; 2003; J Psychiatric Research 37: 267-275

**Herwig U, Padberg F, Unger J, Spitzer M, Schönfeldt-Lecuona;** Transcranial magnetic stimulation in therapy studies: examination of the reliability of “standard” coil positioning by neuronavigation; 2001; Biol Psychiatry 50: 58-61

**Herwig U, Strapi P, Schönfeldt-Lecuona C;** Using the international10-20 EEG system for positioning of transcranial magnetic stimulation; 2003; Brain Topography 16: 95-99

**Höflich G, Kaspr S & al;** Application of transcranial magnetic stimulation in treatment of drug-resistant major depression: a report of two cases; 1993; Human Psychopharmacol 8: 361-365

**Holtzheimer PE 3rd**, **Russo J, Avery DH**; A meta-analysis of repetitive transcranial magnetic stimulation in the treatment of depression; 2001; Psychopharmacol Bull 35:149-69

**Janicak PG, Dowd SM, Martis B & al** ; Repetitive transcranial magnetic stimulation versus electroconvulsive therapy for major depression: preliminary results of a randomized trial ; 2002 ; Biol Psychiatry 51 : 659-667

**Jouvent R, Widlöcher, Lecrubier Y**; Echelle De Ralentissement*,* 1983

**Kearns NB, Cruickhank CA, Riley SA, Shaw SP, Snaith RP;** A comparison of depression rating scales ; 1982 ; Br J Psychiatry 141 : 45-49

**Kimbrell TA, Dunn RT, Georges MS, Danielson AL, Willis MW, Repella JD & al**; Left prefrontal repetitive transcranial magnetic stimulation and regional glucose metabolism in normal volunteers; 2002; Psychiatry Res 115: 101-113

**Kleinjung T, Steffens T, Langguth B & al**; Treatment of chronic tinnitus with neuronavigated repetitive transcranial magnetic stimulation; 2006; HNO 54: 439-444

**Knecht, Sommer J, Deppe M, Steinsträter O**; Scalp position and efficacy of transcranial stimulation; 2005; J Clin Neurophysiol 116: 1988-1993

**Kozel FA, George MS**; Meta-analysis of left prefrontal repetitive transcranial magnetic stimulation (rTMS) to treat depression; 2002; J Psychiatr Pract 8: 210-215

**Lecrubier Y, Sheehan DV & al;** *MINI* 5.0.0 French version, DMS-IV; August 1998

**Li W, Yang Y, Ye Q, Yang B, Wang Z;** Effect of chronic and low-frequency repetitive transcranial magnetic stimulation on spacial memory in rats; 2007; Brain Res Bull 71: 493-500

**Li X, Nahas Z, Kozel FA, Anderson B, Bohning DE, Georges MS**; Acute left prefrontal transcranial magnetic stimulation in depressed patients is associated with immediately increased activity in prefrontal cortical as well as subcortical regions; 2004; Biol Psychiatry 55: 882-890

**Lisanby SH, Maddox JH, Prudic J, Devanand DP, Sackeim HA**; The effects of electroconvulsive therapy on memory of autobiographical and public events; 2000; Arch Gen Psychiatry 57: 581-590

**Lontis ER, Voigt M, Struik JJ**; Focality assessment in transcranial magnetic stimulation with double and cone coils, 2006 ; J Clin Neurophysiol 23 : 462-471

**Loo CK, Mitchell PB** ; A review of the efficacy of transcranial magnetic stimulation (TMS) treatment for depression, and current and future strategies to optimize efficacy ; 2005 ; J of Affective Disorders 88 : 255-267

**Machii K, Cohen D, Ramos Estebanez C, Pascual-Leone A** ; Safety of rTMS to non-motor cortical areas in healthy participants and patients ; 2006 ; Clin Neurophysiol 117 : 455-471

**MacLoughlin DM, Mogg A Eranti S & al;** The clinical effectiveness and cost of repetitive transcranial magnetic stimulation versus electroconvulsive therapy in severe depression: a multicentre pragmatic randomised controlled trial and economic analysis; 2007 ; Health Technol Assess 11 : 1-54

**McNamara B, Ray JL, Arthurs OJ, Boniface S**. Transcranial magnetic stimulation for depression and other psychiatric disorders.2001; Psychol Med: 31:1141-6.

**Maeda F, Keenan JP, Tormos JM, Topka H, Pascual-Leone A**; Interindividual excitability of the modulatory effects of repetitive transcranial stimulation on cortical excitability ; 2000 ; Experimental Brain Research 133 : 425-430

**Martin JL, Barbanoj MJ, Schlaepfer TE, Thompson E, Pérez V, Kulisevsky J;** Repetitive transcranial magnetic stimulation for the treatment of depression. Systematic review and meta-analysis. 2003; Br J Psychiatry: 182:480-91

**Martis B, Alam D, Dowd S & al** ; Neurocognitive effects of repetitive transcranial magnetic stimulation in severe major depression; 2003; Clin Neurophysiol 114: 1125-1132

**Mayberg HS, Liotti M, Brannan SK & al;** Reciprocal limbic-cortical function and negative mood: converging PET finding in depression and normal sadness; 1999; Am J Psychiatry. 156:675-682

**Milner B, Petrides M***; Behavioral effects of frontal-lobe lesions in man*; 1984; Trends Neurosci 7: 403-407

**Nahas Z, Lomarev M, Roberts DR & al**; Unilateral left prefrontal transcranial magnetic stimulation produces intensity-dependent bilateral effects as mesured by interleaved BOLD fMRI; 2001 ; Biol Psychiatry 50 : 712-720

**Nikolaus S, Larish R, Beu M, Vosberg H, Muller-Gartner HW;** Diffuse cortical reduction of neuronal activity in unipolar major depression: a retrospective analysis of 337 patients and 321 controls; 2000; Nucl Med Commun 21 (12): 1119-1125

**Padberg F, George MS**; Repetitive transcranial magnetic stimulation of the prefrontal cortex in depression; 2009; Exp Neurol 219: 2-13.

**Padberg F, Zwanzger P, Keck ME & al** ; Repetitive transcranial magnetic stimulation (rTMS) in major depression : relation between efficacy and stimulation intensity ;2002 ; Neuropsychopharmacology 27 :638-645

**Pascual-Leone A, Dolores-Catala M, Pascual-Leone PA**; lateralized effect of rapid rate transcranial magnetic stimulation of the prefrontal cortex on mood; 1996; Neurology 46: 499-502

**Pascual-Leone A, Houser CM, Reese K & al** ; *Safety of rapid-rate transcranial magnetic stimulation in normal volunteers* ; 1993 ; Electroencephalogr Clin Neurophisiol 89 : 120-130

**Pascual Leone A, Valls-sole J, Wassermann EM, Hallett M**; *Responses to rapid-rate transcranial magnetic stimulation of the human motor cortex*; 1994; Brain 117 : 847-858

**Paus T, Barrett J**; Transcranial magnetic stimulation of the human frontal cortex: implication for repetitive TMS treatment of depression**;** 2004 ; J Psychiatry Neurosci 29 : 268-279

**Paus T, Jech R, Thompson JC, Comeau R, Peters T, Evans AC**; Transcranial magnetic stimulation during positon emission tomography: a new method for studying connectivity of the cerebral cortex; 1997; J Neurosci 17: 3178- 3184

**Pridmore S, Bruno R, Turnier-Shea Y, Reid P, Rybak M** ; Comparison of unlimited numbers of rapid transcranial stimulation and ECT treatment sessions in major depressive episode ; 2000 ; Int J Neuropsychopharmacol 3 :129-134

**Rajkowska G, Goldman-Rakic PS**; Cytoarchitectonic definitionof prefrontal areas in the normal human cortex: Variability in location of area 9 and 46 and relationship to the Talairach coordinate system; 1995; Cereb Cortex 5: 323-337.

**Rogers MA, Kasai K, Koji M;** Excecutive and prefrontal dysfunction in unipolar depression: a review of neuropsychological and imaging evidence; 2004; Neurosci Res 50 :1-11

**Rossini D, Magri L, Lucca A, Gordani S, Smeraldi E, Zanardi R** ; Does rTMS hasten the response to escitalopram, sertraline, or venlafaxine in patients with major depressive disorder? A double-blind, randomized, sham-controlled trial ; 2005 ; J Clin Psychiatry 66 : 1569-1575

**Rumi DO, Gattaz WF, Rigonatti SP & al**; Transcranial magnetic stimulation accelerates the antidepressant effect of amitriptyline in severe depression: a double-blind placebo-controlled study; 2005 ; Biological Psychiatry 57 : 162-166

**Sakkas PP, Mihalopoulou P, Moutzouhou P & al**; Induction of mania by rTMS: report of two cases; 2003; Eur Psychiatry 18 : 196-198

**Schönfeldt-Lecuona C, Thielscher A, Freudenmann RW, Kron M, Spitzer M, Herwig U;** Accuracy of stereotaxic positioning of transcranial magnetic stimulation; 2005; Brain Topogr 17: 253-259

**Thase ME & Rush AJ**; When at first you don’t succeed; sequential strategies for antidepressant non responders; 1997; J Clin Psychiatry 58: 23-29

**Tekin S, Cummings JL;** Frontal-subcortical neuronal circuits and clinical neuropsychiatry: an update; 2002; J Psychosom Res 53: 647-654

**Thielscher A, Krammer T**; Electric field properties of two commercial figure-8 coils in TMS: calculation of focality and efficiency; 2004 ; Clin Neurophysiol 115 : 1697-1708

**Vanderhasselt MA, de Raedt R, Baeken C, Leyman L, d’Haenen H**; A single session of rTMS over the left dorsolateral prefrontal cortex influences attentional control in depressed patients, 2007; World J Biol Psychiatry 17: 1-9

**Wassermann EM**; Risk and safety of repetitive transcranial magnetic stimulation: report and suggested guidelines from the International Workshop on the safety of repetitive transcranial magnetic stimulation June 5-7 1996; 1998 ; Electroencephalog Clin Neurophysiology 108 : 1-16

**West j, Fitzpatrick J, Wang M, Dawant B, Maurer C, Kessler R, Maciunas R.** Retrospective inter modality registration techniques for images of the head: surface-based versus volume-based; TMI, 1999, (2) 18 144-150.

**Wirtz CR, Knauth M, Hassfeld S & al:** Neuronavigation-first experiency with three different available systems; 1998; Zentralbl Neurochir 59: 14-22

# ANNEXES

## ANNEXE 1 : FORMULAIRE D’INFORMATION

## ANNEXE 2 : Formulaire de consentement

## ANNEXE 3

**CHECK LISTE DES CRITERES D’INCLUSION ET DE NON INCLUSION**

|  | | OUI | NON |
| --- | --- | --- | --- |
| I  N  C  L  U  S  I  O  N | Episode dépressif majeur (DSM IV) |  |  |
|  | Droitiers |  |  |
|  | Agé de plus de 18 ans moins de 65 ans |  |  |
|  | Traitement stable depuis 3 semaines |  |  |
|  | Score MADRS > 21 |  |  |
| E  X  C  L  U  S  I  O  N | Dépression avec caractéristiques psychotiques |  |  |
|  | Malade hospitalisé sous contrainte ou mesures de protection |  |  |
|  | Comorbidité sur l’axe 1 de schizophrénie ou pathologie addictive |  |  |
|  | Antécédents médicaux ou matériel prothétique |  |  |
|  | Examen complémentaires anormaux (EEG) radio du crâne) |  |  |
|  | Si femme : grossesse ou allaitement |  |  |
|  | Résistance définie par le stade V de classification résistante de Thase et Rush |  |  |
|  | | | |
| Le sujet peut être inclus dans le protocole thérapeutique | |  |  |

## ANNEXE 4

**CLASSIFICATION DE THASE & RUSH (1997)**

| Stage I | Failure of at least one adequate trial of one major class of antidepressant |
| --- | --- |
| Stage II | Stage I resistance plus failure of adequate trial of an antidepressant in a distinctly different class from that used in stage I |
| Stage III | Stage II resistance plus failure of an adequate trial of TCA |
| Stage IV | Stage III resistance plus failure of an adequate trial of MAOI |
| Stage V | Stage III resistance plus failure of an adequate trial of bilateral  ECT |
| TCA Tricyclic antidepressant, MAOI MonoAmine Oxydase Inhibitor, ECT ElectroConvulsiveTherapy | |

Thase ME & Rush AJ; When at first you don’t succeed; sequential strategies for antidepressant non responders; 1997; J Clin Psychiatry 58: 23-29

1. Vision, Action et Gestion d'Informations en Santé : L'Unité/Projet VisAGeS - U746 est une équipe de recherche reconnue conjointement par l'INSERM et l'INRIA, qui travaille dans le domaine du développement de nouveaux algorithmes de traitement informatique des images médicales et des interventions assistées par ordinateur. [↑](#footnote-ref-2)
2. INRIA : Institut National de Recherche en Informatique et Automatique [↑](#footnote-ref-3)
