# Supplementary material for: Repetitive Transcranial Magnetic Stimulation targeted with MRI based neuro-navigation in major depressive episode: a double-blind, multicenter randomized controlled trial
Source: PLoS One. 2025 May 27;20(5):e0317597. doi: 10.1371/journal.pone.0317597 (PMC12111610; doi:10.1371/journal.pone.0317597)
Supplement: S2 File — (DOCX) [file pone.0317597.s002.docx]

**English translation of the protocol (summary)**

Done with deepL on 06/01/2025

MAIN OBJECTIVE

In view of the data in the literature, the aim of this project is to evaluate, by means of a randomised double-blind multicentre study in two parallel groups, the therapeutic superiority of localised stimulation of the left dorsolateral prefrontal cortex (DLPFC) by a neuronavigator compared with stimulation of the left dorsolateral prefrontal cortex (DLPFC) located in a standard manner in the treatment of isolated or recurrent depressive states.

Our hypothesis is that localised stimulation of the CPFDL produces a clinical response that is qualitatively and quantitatively superior to less precise stimulation.

SECONDARY OBJECTIVES

To test the superiority of the feeling of therapeutic improvement in patients stimulated with the neuronavigation system compared with patients stimulated with the standard method using the Beck Depression Inventory (BDI).

To test the superiority of psychomotor improvement in patients stimulated with the neuronavigation system compared with patients stimulated with the standard method, using the Depression and Slowness Scale (DSS).

PRIMARY ENDPOINT

The primary endpoint was a clinical response defined as a halving of the MADRS score at D44, following treatment with TMS

treatment and the follow-up period.

SECONDARY ENDPOINTS

One of the secondary endpoints is the percentage of patients in clinical remission at D14 and D44.

Clinical remission was defined as a MADRS score of less than or equal to 8.

The percentage of responding patients at D14 is also a secondary endpoint.

Perceived clinical improvement was also assessed via the change in the BDI scale after treatment at D14 and during follow-up (D44) in each group.

Psychomotor slowing was assessed via changes in the ERD scale after treatment and during follow-up in each group.

STUDY METHODOLOGY / DESIGN

Randomised, double-blind, multicentre study of patients meeting the diagnostic criteria for isolated or recurrent Major Depressive Episode, divided into :

. a group of 60 patients receiving empirical anatomical identification of the target and stimulation not assisted by a neuronavigator

. a group of 60 patients receiving MRI target location and neuronavigator-guided stimulation.

The stimulation parameters used will be as follows: 10 sessions, 20Hz, 110% of motor threshold, 1600 pulses/session.

Primary endpoint :

Improvement in Montgomery and Adsberg Rating Scale (MADRS; Montgomery et al, 1979) score before and after transcranial magnetic stimulation as well as 30 days after the end of stimulation.

Secondary endpoints :

Changes in scores on the Beck Depression Inventory (BDI) and Slowness Scale (SRS).

Patients were assessed by the same assessor before the first session, after the 10 sessions and one month after the end of the sessions. The stimulation sessions are carried out by an operator who is independent of the assessor.

The investigating centres involved will be CHU de Rennes/CHGR, CHU de Brest, EPSM Saint-Avé, CESAME Angers, EPSM Quimperlé, Clinique St Laurent (Rennes). Patients will be recruited over a 2-year period.

This study will be carried out after obtaining the opinion of the CPP, with due respect for individuals (information, informed consent and confidentiality). It will be conducted in accordance with the rules of good clinical practice.

SUBJECT INCLUSION CRITERIA

Right-handed volunteers over 18 and under 65 years of age:

- Have signed a free and informed consent to participate in the study.

- Diagnosed as having a recurrent or isolated major depressive episode (MDE) according to DSM IV criteria (A.P.A. 1994).

- On unchanged antidepressant treatment for 3 weeks.

- MADRS score ≥ 21

Patients' antidepressant treatment must not have been modified in the 3 weeks prior to inclusion to avoid confounding bias between antidepressant treatment and rTMS treatment. Benzodiazepine treatments should be discontinued insofar as they can be replaced by other anxiolytic treatments and reduce cortical excitability. Thymoregulatory treatments, although they also reduce cortical excitability, should not be stopped as they cannot be substituted.

CRITERIA FOR NON-INCLUSION OF SUBJECTS

- Depression with psychotic features

- Co-morbid diagnosis according to Axis I (DSM IV) of schizophrenia, dependence (or abuse) on alcohol and/or another substance (lifetime)

- Patient meeting the criteria for resistant depression as defined by stage V of the Thase & Rush classification (see appendix 4)

- Patients hospitalised under duress or under legal protection (guardianship, curatorship)

- Patient presenting a high suicide risk (item 10 MADRS > 3) in the absence of hospitalisation

- Contraindication to MRI or rTMS: personal history of comitial seizure, neurological or neurosurgical pathologies, metallic prosthetic material or foreign bodies (pacemaker, intraocular ferromagnetic material)

- Patients aged over 65, due to the risk of cortical atrophy.

- Pregnancy.

DEVICES / STRATEGIES / PROCEDURES

Localised stimulation of the left CPFDL by a neuronavigator is compared with stimulation of the left CPFDL located in the standard way in the treatment of isolated or recurrent depressive states.

Whichever arm patients are randomised to, all must have undergone the 3D MRI necessary for neuronavigation.

- If a patient has already undergone 3D MRI during their usual follow-up, then this will be used without repeating it;

- If a patient has never had 3D MRI, then it must be carried out within 15 days of randomisation’.

After being randomised, patients are assigned to one of the following treatments:

- high-frequency rTMS (20Hz) applied to the left CPFDL identified by neuronavigation

- high-frequency rTMS (20Hz) applied to the left CPFDL identified by the standard ‘5 cm’ method.

The first TMS session must be performed no later than 15 days after randomisation. This defines D0 of the study.

The treatment is delivered by a clinician other than the investigator-cotator, informed by the ICC of the randomisation arm.

The MADRS, BDI and ERD scales were administered by the investigator-indicator blinded to the treatment arm, before the first TMS session.

After the 10 rTMS treatment sessions, patients entered a follow-up phase during which they were assessed twice: after the last session (D14) and 1 month after the last session (D44).

Antidepressant treatment, stable for at least 3 weeks, will only be modified in the event of a therapeutic emergency. Any changes will be recorded in the observation book.

In the event of major suicidal risk (item 10 MADRS> 3), patients will be hospitalised in accordance with good clinical practice.

NUMBER OF PATIENTS

The question asked is formulated bilaterally.

The expected rate of improved patients (MADRS reduced by 50%) in the ‘standard stimulation’ arm is 35%. The benefit expected with neuronavigation should make it possible to increase this rate to 70%. If we want to have the statistical means to detect an absolute increase of 35% in the ‘neuronavigation’ arm, we need to include 60 subjects per arm to guarantee a power of 95% in a test carried out with a risk of error of alpha = 5%.

The total number of subjects required is therefore 120.
