# Supplementary material for: Repetitive Transcranial Magnetic Stimulation targeted with MRI based neuro-navigation in major depressive episode: a double-blind, multicenter randomized controlled trial
Source: PLoS One. 2025 May 27;20(5):e0317597. doi: 10.1371/journal.pone.0317597 (PMC12111610; doi:10.1371/journal.pone.0317597)
Supplement: S3 File — (PDF) [file pone.0317597.s003.pdf]

**TMS**	Version 1.0 du 14/11/2018

Rédacteur : Valérie Turmel

Le plan d'analyse statistique est écrit selon le protocole V12.0 daté du 14/09/15.

**Méthodologiste(s) :**	**Investigateur(s) coordonnateur (s) :**
NAUDET Florian<br>Unité de soins psychiatriques CHU de Rennes<br>Tél. : 02.99.28.25.68<br>Mail : florian.naudet@chu-rennes.fr<br><br>En collaboration avec REYMAN Jean-Michel	Pr Dominique Drapier<br>Service de psychiatrie<br>Centre Hospitalier Guillaume Regnier<br>108 AV. DU GENERAL LECLERC - B.P. 60321<br>35703 Rennes<br>Mail : dominique.drapier@univ-rennes1.fr<br><br>Et<br><br>Pr Bruno Millet<br>Groupe Hospitalier Pitié-Salpêtrière, Service de Psychiatrie Adulte, Pavillon La Force,<br>47 boulevard de l'Hôpital,<br>75651 Paris Cedex 13.<br>Mail : b.millet@aphp.fr

1. Rappel sur le protocole

1.1. Objectifs

➢ **Objectif principal**

Au vues des données de la littérature, l'objectif de ce projet est d'évaluer par une étude multicentrique randomisée en double aveugle sur deux groupes parallèles, la supériorité thérapeutique de la stimulation localisée du cortex préfrontal dorsolatéral (CPFDL) gauche par un neuronavigateur par rapport à la stimulation du cortex préfrontal dorsolatéral (CPFDL) gauche repéré de manière standard dans le traitement des états dépressifs isolés ou récurrents.
L'hypothèse est qu'une stimulation localisée du CPFDL permet une réponse clinique qualitativement et quantitativement supérieure à une stimulation moins précise.

➢ **Objectifs secondaires**

Les objectifs secondaires sont :
- Tester la supériorité du ressenti de l'amélioration thérapeutique chez les patients stimulés avec le système de neuronavigation par rapport aux patients stimulés avec la méthode standard au moyen du Beck Depression Inventory (BDI)
- Tester la supériorité de l'amélioration du plan psychomoteur chez les patients stimulés avec le système de neuronavigation par rapport aux patients stimulés avec la méthode standard au moyen de l'Echelle de Dépression et de Ralentissement (EDR)

1.2. Critères de jugement

➢ **Critère d'évaluation principal**

Le critère de jugement principal est l'obtention d'une réponse clinique définie par une diminution de la moitié du score de la MADRS à J44, à l'issue du traitement par TMS et de la période de suivi.

➢ **Critères d'évaluation secondaires**

- Un des critères de jugement secondaire est le pourcentage de patients présentant une rémission clinique à J14 et J44. La rémission clinique est définie par un score à la MADRS inférieur ou égal à 8.
- Le pourcentage de patients répondeurs à J14 est également un critère secondaire.
- Le ressenti de l'amélioration clinique est également évalué via la variation de l'échelle BDI après le traitement à J14 et au cours du suivi (J44) dans chaque groupe.
- Le ralentissement psychomoteur est évalué via la variation de l'échelle ERD après le traitement et au cours du suivi dans chaque groupe.

1.3. Design

- **Plan expérimental :**

Etude multicentrique randomisée en double aveugle, contrôlé, de patients répondant aux critères diagnostiques d'Episode dépressif Majeur isolé ou récurrent, répartis en 2 groupes :

- un groupe de 60 patients bénéficiant d'un repérage anatomique empirique de la cible et d'une stimulation non assistée par un neuronavigateur
- un groupe de 60 patients bénéficiant d'un repérage de la cible en IRM et d'une stimulation guidée par neuronavigateur.

Les paramètres de stimulation utilisés seront les suivants: 10 séances, 20Hz, 110 % du seuil moteur, 1600 pulses/séance.

- **Nombre de sujets :**

La question posée est en formulation bilatérale.
Le taux attendu de patients améliorés (MADRS diminuée de 50%) dans le bras « stimulation standard » est de 35%. Le bénéfice attendu avec la neuronavigation doit permettre d'augmenter ce taux à 70% Si l'on veut se donner les moyens statistiques de détecter une augmentation absolue de 35% dans le bras « neuronavigation » il faut inclure 60 sujets par bras pour garantir une puissance de 95% dans un test réalisé avec un risque d'erreur α = 5%.]
Le nombre total de sujets nécessaires est donc de **120** (60 par groupe)

- **Déroulement de l'étude**

Recrutement des participants :

Les 8 centres investigateurs impliqués sont les suivants :

(1) CHGR / CHU de Rennes
(2) Centre de Santé Mentale Angevin, Angers
(3) CHU de Brest
(4) Centre hospitalier Quimperlé
(5) Etablissement Public de Santé Mentale Saint-Avé
(6) Clinique St Laurent, Rennes
(7) CH Laborit, Poitiers
(8) A APHP – Pitié-Salpêtrière

Au final, 4 centres ont inclus : (1) CHGR / CHU de Rennes, (5) Etablissement Public de Santé Mentale Saint-Avé, (7) CH Laborit Poitiers, (8) A APHP – Pitié-Salpêtrière

Randomisation

La randomisation est stratifiée selon le centre.
Après vérification des critères d'inclusion et de non inclusion, l'investigateur procède à la randomisation du patient. Elle s'effectue sur l'e-CRF de l'étude.Le logiciel attribut au malade le premier numéro disponible et informe uniquement le clinicien réalisant les rTMS du bras de randomisation du patient. **Le patient est randomisé lors de la visite d'inclusion.**

Actions et données recueillies

	Inclusion/ Randomisation	J0	J14	J44
Consentement du patient/proche	x			
IRM	x			
Vérification des critères d'inclusion et non inclusion	x			
Données socio-démographique	x			
Antécédents médicaux et chirurgicaux significatifs	x			
Evaluation psychiatrique (MINI 5.0)	x			
Donnée concernant la pathologie thymique	x			
Echelle MADRS	x	x	x	x
Echelle ERD		x	x	x
Echelle BDI		x	x	x

Schéma du protocole :

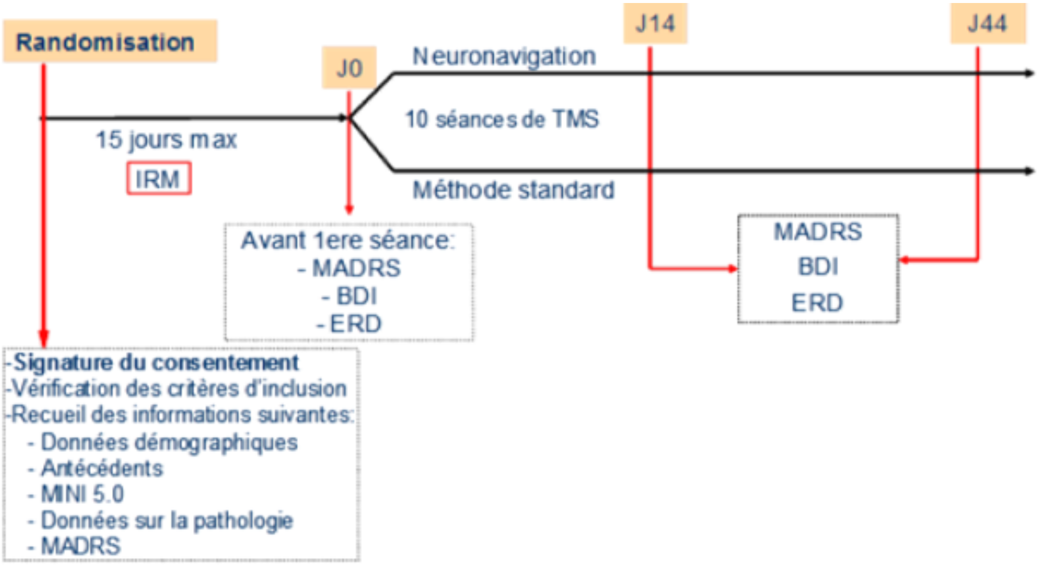

Les patients sont évalués par un cotateur avant la première séance, après les 10 séances et un mois après la fin des séances par ce même cotateur. Les séances de stimulations sont réalisées par un opérateur indépendant du cotateur

Durée de l’étude :

Date de début : janvier 2013
Période de recrutement : 4 ans
Durée de suivi : 59 jours
Durée totale de l’étude : 52 mois
Date de fin d'étude : avril 2017

1.4. Analyse prévue

Cf. 1.2 + *« Extrait protocole » - Page 24 et 26*

L’analyse statistique portera sur tous les patients randomisés et évalués ayant donné leur consentement (Analyse en intention de traiter).

A noter : Il sera proceder à 2 types d’analyses

- ❖ Analyse en intention de traiter
  « Les éventuels patients devant recevoir en cas d'urgence une modification de leur traitement habituel seront considérés comme échecs sur le critère de jugement principal et les critères secondaires qualitatifs c'est-à-dire comme non répondeurs et n'étant pas en rémission. Ils seront pris en compte dans l'analyse, dans le bras où ils ont été randomisés, en respect du principe de l'analyse en intention de traiter. »
  Population ITT = 102 sujets
- ❖ Analyse de sensibilité (population en per-protocole)
  « Il est prévu de réaliser une analyse de sensibilité sur la population en per protocole. Celle-ci se définit comme la population ayant reçu le traitement à l’étude conformément au protocole et ayant bénéficié d’une localisation automatique pour les patients neuronavigués. »
  Population per-protocole = 102 sujets
  – 27 sujets non conformes au protocole et/ou n’ayant pas bénéficié d’une localisation automatique pour les patients neuronavigués(cf. rapport qualité des données).
  Soit population per-protocole = 75 sujets

- ➢ **Analyse descriptive par groupe**

Une première analyse descriptive globale et par groupe est réalisée. Elle comporte des estimations ponctuelles, nombres et pourcentages pour les variables qualitatives, moyennes, écart-types, médianes et intervalles interquartiles pour les variables quantitatives. La normalité de la distribution des variables quantitatives est vérifiée.

- ➢ **Comparaison des groupes en fonction du traitement reçu**

Comparabilité des groupes à l’inclusion
Test du Chi² ou test exact de Fisher si nécessaire pour les variables qualitatives
Test t de Student ou test de Wilcoxon si nécessaire pour les variables quantitatives.

Analyse sur le critère principal
Test du chi2 et analyses ajustées sur les principales variables pronostiques ou déséquilibrées à l’inclusion à l’aide d’un modèle de régression logistique.

Analyses sur les autres critères
Test du Chi² ou test exact de Fisher si nécessaire pour les variables qualitatives
Test t de Student ou test de Wilcoxon si nécessaire pour les variables quantitatives.

- **Analyse des évènements indésirables**

Les éventuels évènements indésirables sont codés selon la classification MedDRA et font l'objet d'une analyse descriptive. Cf. rapport de sécurité de TMS. Les événements indésirables seront décrits selon leur fréquence de survenue, leur gravité et leur imputabilité à l'un ou à l'autre des traitements de l'étude.

2. Changements par rapport au protocole

L'analyse en ITT porte sur les sujets pour lesquels le critère principal est évalué. 89 sujets ont le critère principal sur les 102 patients randomisés. La méthode LOCF est appliqué sur 3 patients (affectation de la MADRS J14 à la MADRS à J44)) portant à un effectif de 92 sujets évaluables sur le critère principal

3. Populations analysées

3.1. Nombre de sujets inclus

105 patients randomisés

102 patients inclus (3 exclus)

3.2. Populations

Cf. Flow chart de l'étude

Populations :

- Inclus randomisés : N=102 (50 patients dans le groupe 1 – Neuronavigateur ; 52 patients dans le groupe 2 – Standard)

- Pour l'analyse ITT d'efficacité, 13 patients sont non évaluables, d'où N=89.
En appliquant la méthode LOCF : 10 patients ne sont pas évaluables, d'où N=92

- Pour l'analyse de sensibilité per-protocole, 27 patients sont non évaluables, d'où N=75
Rappel : l'analyse de sensibilité en per protocole se définit comme la population ayant reçu le traitement à l'étude conformément au protocole et ayant bénéficié d'une location automatique pour les patients neuronavigués. *(cf. protocole)*

- Pour l'analyse de tolérance, le recueil des évènements indésirables se fait :
. lors du suivi (période J0 à J44)
. lors des séances de rTMS :
L'analyse de la tolérance porte sur la population incluse randomisée, d'où N=102

TMS	Version 1.0 du 14/11/2018

TMS - Flow Chart

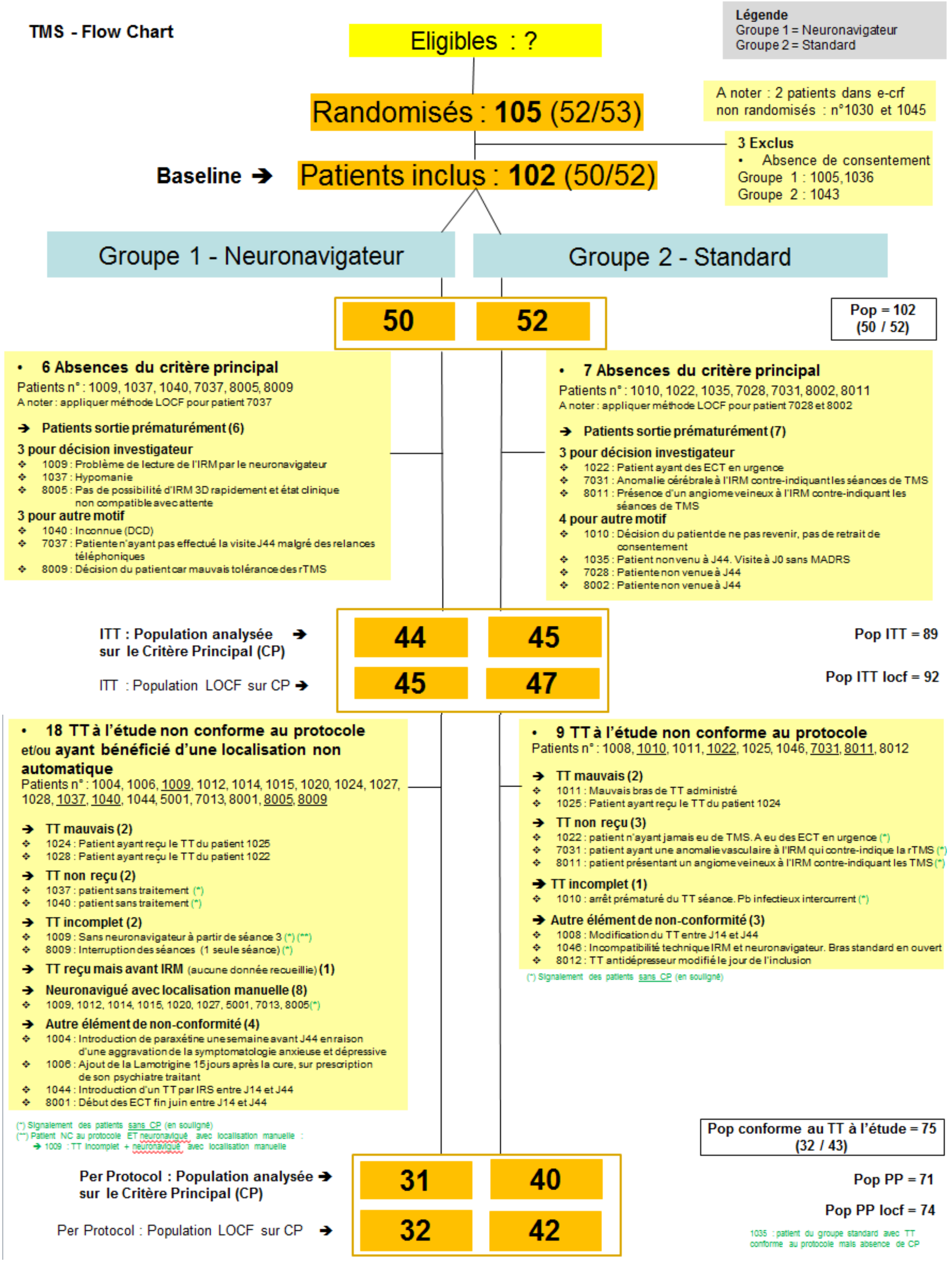

4. Généralités

- Version du logiciel statistique utilisé : le logiciel SAS V9.4 (SAS Institute, USA)
- Seuil de significativité de 5%
- Présentation par groupe de traitement

5. Analyse des critères

5.1. Description de la population à l'inclusion

- Caractéristiques démographiques et cliniques à l'inclusion :
  - ✓ Age
  - ✓ Sexe
  - ✓ Situation familiale
  - ✓ A un ou des enfants
  - ✓ Niveau d'éducation
  - ✓ Activité professionnelle
- Evaluation psychiatrique standardisée des critères d'inclusion et de non-inclusion
  - ✓ Score MADRS total
  - ✓ Score MADRS item 10
- Données concernant la pathologie thymique (épisode actuel)
  - ✓ Durée de l'épisode Dépressif Majeur actuel
  - ✓ Stade de Thase et Rush
- Données concernant la pathologie thymique (épisodes antérieurs éventuels et comorbidités)
  - ✓ Type de trouble de l'humeur
  - ✓ Année de diagnostic du premier épisode thymique
  - ✓ Type du premier époside thymique
  - ✓ Nombre d'épisodes dépressifs antérieurs
  - ✓ Nombre d'épisodes hypomaniaques et maniaques antérieurs
  - ✓ Antécédent de tentaive de suicide
  - ✓ Antécédent de trouble anxieux
  - ✓ Traitement antérieur par TMS
- Données concernant la pathologie thymique (antécédents familiaux)
  - ✓ Antécédent familial au premier degré de trouble de l'humeur
  - ✓ Antécédent familial au premier degré de suicide
- Traitement médicamenteux début avant l'étude
- Fin d'essai
  - ✓ Essai conforme au protocole
  - ✓ Traitement à l'essai arrêté prématurément (si essai non conforme au protocole)
    - o Motif et décision
    - o Description si motif pour EING et EIG
  - ✓ Sortie d'essai prématurément (si essai non conforme au protocole)
    - o Motif et précision

5.2. Analyse de l'efficacité de stimulations par méthode neuronavigateur versus méthode standard

- Analyse du critère d'évaluation principal
  - ✓ % de patient ayant une réponse clinique à J44, la réponse clinique étant définie par une diminution de la moitié du score de la MADRS entre J0 et J44

- Analyse des critères d'évaluation secondaires
  - ✓ % patients ayant une réponse clinique à J14, la réponse clinique étant définie par une diminution de la moitié du score de la MADRS entre J0 et J14
  - ✓ % de patients présentant une rémission clinique à J14 et J44, la rémission clinique étant définie par un score MADRS inférieur à 8.
  - ✓ Variation de l'échelle BECK/BDI à J14 et à J44, permettant d'évaluer le ressenti de l'amélioration clinique
  - ✓ Variation de l'échelle ERDà J14 et à J44, permettant d'évaluer le ralentissement psychomoteur

5.3. Analyse de la tolérance

- ✓ Evènements indésirables
  - o en terme de nombre de patients
  - o en terme de nombre d'EI (1 enregistrement dans la base e-crf)
- ✓ Analyse selon la classification MEDRA

5.4. Analyse complémentaire

- ✓ Analyse de sensibilité per protocole

6. Historique des modifications

Date	Nom du document	Version	Modification/Contexte
14/11/18	PAS_NOMETUDE_181114_V1	V1	Première version
