# Supplementary material for: Repetitive Transcranial Magnetic Stimulation targeted with MRI based neuro-navigation in major depressive episode: a double-blind, multicenter randomized controlled trial
Source: PLoS One. 2025 May 27;20(5):e0317597. doi: 10.1371/journal.pone.0317597 (PMC12111610; doi:10.1371/journal.pone.0317597)
Supplement: Attachment — Submitted filename: Comments to Authors.pdf [file pone.0317597.s004.pdf]

Comments to Authors

This multicenter, double-blind randomized controlled trial compared MRI-based neuronavigation with the standard 5 cm method for rTMS targeting in major depressive disorder. While the study findings did not support the authors' hypothesis, the results remain valuable in advancing our understanding of rTMS targeting. To enhance the quality of this manuscript, several major and minor concerns should be addressed.

Major Concerns

- The title refers to "major depressive disorder," and the recruitment criteria cite " major depressive disorder according to DSM-IV criteria in a current depressive episode." However, Table 1 indicates that approximately 60% of participants were diagnosed with persistent depressive disorder and 20% with bipolar disorder. Since persistent depressive disorder and bipolar disorder are distinct from major depressive disorder, the title should be revised accordingly.
- The term "persistent depressive disorder" is not part of DSM-IV terminology, which uses "dysthymic disorder." The authors should clarify whether they used DSM-IV or DSM-5 criteria.
- If participants with persistent depressive disorder were indeed diagnosed solely with dysthymic disorder, this implies that these individuals may not have experienced a major depressive episode. While the recruitment threshold of ≥21 on the Montgomery-Åsberg Depression Rating Scale appears appropriate, the diagnostic criteria and severity thresholds need to align clearly.

Minor Concerns

Abstract

- The authors report relative risks with 95% confidence intervals (CIs) but do not describe the statistical methods in the Methods section.
- Adverse event rates are described as "similar," yet no statistical results are reported for this comparison. Statistical tests should be included for consistency.

Introduction

- The authors aim to "reproduce the findings from Fitzgerald et al." However, their rTMS protocol (20 Hz with 40 pulses per train, 80 trains, inter-train interval of 10 seconds, 110% resting motor threshold, and 10 sessions) differs significantly from that

used in Fitzgerald et al. These differences should be explained and acknowledged in the Introduction.

Methods

- The rationale for using 20 Hz rather than the more common 10 Hz protocol should be provided. While 20 Hz is not inherently problematic, justification for its selection is necessary.
- Details on statistical methods are insufficient. The authors should specify how they calculated relative risks, absolute risk differences, and 95% CIs, in addition to the Chi-squared tests and t-tests already described.

Results

- Statistical results for safety data should be reported in greater detail, including relative risks or absolute risk differences, similar to the efficacy outcomes.
- Given the inclusion of bipolar disorder patients, subgroup analyses should be conducted and reported separately for this population. Previous literature on rTMS for bipolar disorder is limited, and such analyses would add value, particularly in mixed cohorts of major depressive disorder and bipolar disorder patients.

Discussion

- The authors state that "the limited number of sessions prevented a more pronounced differentiation between groups." Previous research (e.g., Hsu TW, Neurosci Biobehav Rev, 2024 Jul;162:105704) supports the idea that a greater number of sessions can enhance overall efficacy. The authors are encouraged to elaborate further on this point.
- The recruitment of ~20% bipolar disorder patients warrants additional discussion. A recent meta-analysis (Hsu CW, Neurosci Biobehav Rev, 2024 Jan;156:105483) suggests that high-frequency stimulation of the left DLPFC may not be optimal for bipolar disorder, potentially impacting overall efficacy. The authors should explore this issue in greater depth.
