# Supplementary material for: Repetitive Transcranial Magnetic Stimulation targeted with MRI based neuro-navigation in major depressive episode: a double-blind, multicenter randomized controlled trial
Source: PLoS One. 2025 May 27;20(5):e0317597. doi: 10.1371/journal.pone.0317597 (PMC12111610; doi:10.1371/journal.pone.0317597)
Supplement: Attachment — Submitted filename: RESPONSES.docx [file pone.0317597.s007.docx]

Dear Editors of *PLOS ONE*,

We respectfully appeal the decision to reject our manuscript, as we believe it meets the criteria for publication in *PLOS ONE*. Our study, a randomized controlled trial, presents original findings on the use of MRI-based neuronavigation for rTMS targeting in major depressive episodes. In response to the reviewers’ feedback, we have carefully addressed each of the points raised. We are confident that these revisions have substantially improved the manuscript and that it now aligns with *PLOS ONE*’s standards for publication.

As requested, we have included the following documents for your review:

1. Appeal Request Form
2. Response to Reviewers
3. Track Changes Version of the Manuscript (and, in addition, the Statistical Analysis Plan)

Thank you for considering our appeal. We look forward to your feedback.

Sincerely,
Professor Florian Naudet
(On behalf of all authors)

**Reason for appeal**

The reason provided for the rejection was as follows:

*"After careful consideration, we have decided that your manuscript does not meet our criteria for publication and must therefore be rejected."*

We respectfully disagree with this decision, as we believe our manuscript meets all the PLOS criteria for publication, which can be found here: [PLOS ONE Criteria for Publication](https://journals.plos.org/plosone/s/criteria-for-publication#loc-7). Below, we provide a detailed response to each of these criteria, along with comments on how our manuscript aligns with them:

1. **The study presents the results of original research.**
   Our study is a Randomized Controlled Trial (RCT), presenting original findings.
2. **Results reported have not been published elsewhere.**
   The results of this study have not been published elsewhere.
3. **Experiments, statistics, and other analyses are performed to a high technical standard and are described in sufficient detail.**
   In response to the question, “Has the statistical analysis been performed appropriately and rigorously?” the reviewers’ feedback was as follows:
   - Reviewer #1: Yes
   - Reviewer #2: Yes
4. **Conclusions are presented in an appropriate fashion and are supported by the data.**
   Regarding the question, “Is the manuscript technically sound, and do the data support the conclusions?” the reviewers responded:
   - Reviewer #1: Yes
   - Reviewer #2: Partly

We would like to note that we can easily address Reviewer #2's concerns and provide further clarifications. However, we do not believe these comments alone justify a rejection, especially given our willingness to respond to feedback.

5. **The article is presented in an intelligible fashion and is written in standard English.**
   In response to the question, “Is the manuscript presented in an intelligible fashion and written in standard English?” the reviewers responded:
   - Reviewer #1: Yes
   - Reviewer #2: Yes

We are confident that the manuscript meets the language and clarity standards required for publication.

6. **The research meets all applicable standards for the ethics of experimentation and research integrity.**
   Although there was no specific question about ethics, the reviewers raised no concerns regarding this matter. The study was approved by an ethics committee, and informed consent was obtained from all patients whose data are reported. Furthermore, the study adhered to the Good Clinical Practice guidelines, which are the standard for French academic research.
7. **The article adheres to appropriate reporting guidelines and community standards for data availability.**
   In response to the question, “Have the authors made all data underlying the findings in their manuscript fully available?” the reviewers responded:
   - Reviewer #1: Yes
   - Reviewer #2: Yes

We have ensured that all data underlying our findings are fully available, as required by PLOS Data policies.

Given that our manuscript adheres to all the relevant criteria for publication, we respectfully request a re-evaluation of the decision. Below, we provide more detailed answers to the reviewers’ comments for your consideration.

**Point by point response to the reviewer**

Reviewer #1: This multicenter, double-blind randomized controlled trial compared MRI-based neuronavigation with the standard 5 cm method for rTMS targeting in major depressive disorder. While the study findings did not support the authors’ hypothesis, the results remain valuable in advancing our understanding of rTMS targeting. To enhance the quality of this manuscript, several major and minor concerns should be addressed.


Major Concerns
- The title refers to "major depressive disorder," and the recruitment criteria cite " major depressive disorder according to DSM-IV criteria in a current depressive episode." However, Table 1 indicates that approximately 60% of participants were diagnosed with persistent depressive disorder and 20% with bipolar disorder. Since persistent depressive disorder and bipolar disorder are distinct from major depressive disorder, the title should be revised accordingly.

**We would like to thank the reviewer for this request to clarify our manuscript and agree to revise the term MAJOR DEPRESSIVE DISORDER in MAJOR DEPRESSIVE EPISODE if it is clearer. Indeed, while major depressive disorder is often usen even for bipolar and unipolar depression, the correct term is major depressive episode.**

**We have revised the title accordingly:**

**“Repetitive Transcranial Magnetic Stimulation targeted with MRI based neuro-navigation in major depressive episode: a double-blind, multicenter randomized controlled trial”**

- The term "persistent depressive disorder" is not part of DSM-IV terminology, which uses "dysthymic disorder." The authors should clarify whether they used DSM-IV or DSM-5 criteria.

**We are sorry for the confusion. This is surely a misunderstanding due to an inaccuracy in Table 1 as described in the reviewer’s next comment. Actually all patients had major depressive episode (according DSM-IV) as it was explicitly stated in the text:**

**“All patients were in- and out- patients, aged from 18 to 65, right-handed, suffering major depressive disorder according to DSM-IV criteria in a current mood depressive episode.”**

**We confirm that we used the DSM-IV criteria that were in use in France when we initiated the study.**

**Accordingly, no change has been made to our selection criteria. Please see our response to the next comment for further clarification.**

- If participants with persistent depressive disorder were indeed diagnosed solely with dysthymic disorder, this implies that these individuals may not have experienced a major depressive episode. While the recruitment threshold of ≥21 on the Montgomery-Åsberg Depression Rating Scale appears appropriate, the diagnostic criteria and severity thresholds need to align clearly.

**We thank the reviewer for this comment. As the reviewer knows, dysthymic disorder and major depressive episode are mutually exclusive. Within DSM-IV. As a reminder we have copied below DSM-IV criteria and outlined in red the fact that “the disturbance is not better accounted for by MDD or MDD in partial remission.”**

**DSM-IV**

Name: Dysthymic Disorder

Class: Mood Disorders

Depressed mood for most of the day, for more days than not, as indicated by subjective account or observation by others, for at least 2 years.

Presence while depressed of two or more of the following:

- Poor appetite or overeating
- Insomnia or hypersomnia
- Low energy or fatigue
- Low self-esteem
- Poor concentration or difficulty making decisions
- Feelings of hopelessness

During the 2 year period of the disturbance, the person has never been without symptoms from the above two criteria for more than 2 months at a time.

**The disturbance is not better accounted for by MDD or MDD in partial remission.**

There has never been a manic episode, a mixed episode, or a hypomanic episode and the criteria for cyclothymia have never been met.

The disturbance does not occur exclusively during the course of a chronic psychotic disorder.

The disturbance is not due to the direct physiological effects of a substance (e.g., a drug of abuse or a medication) or a general medical condition.

The symptoms cause clinically significant distress or impairment in important areas of functioning.

**While we did not include patients with dysthymic disorder in this study, the reviewer was right to point what looks like an inaccuracy in Table 1 likely due to an error when translating it from French to English language. We describle the 3 different categories:**

**1/ Isolated unipolar depressive episode**

**2/ Persistent depressive episode**

**3/ Bipolar episode**

**Instead of Persistent depressive episode, category 2 should read recurrent unipolar episode.**

**We have edited the table accordingly.**

**“Recurrent depressive disorder”**

Minor Concerns
Abstract
- The authors report relative risks with 95% confidence intervals (CIs) but do not describe the statistical methods in the Methods section.

**We thank the reviewer for this comment. We have edited the abstract accordingly.**

**“Context: High-frequency (HF) transcranial magnetic stimulation (rTMS) of the left dorsolateral prefrontal cortex (DLPFC) is widely used in Major Depressive Disorder (MDD). Optimization of its efficacy with a neuro-navigation system has been proposed based on a small randomized controlled trial (RCT) supporting a large effect. Method: This evaluator- and patient-blind, multicenter RCT assessed the superiority in terms of efficacy of 10 HF rTMS sessions of the left DLPFC targeted with MRI based neuro-navigation versus similar sessions targeted by the standard 5 cm technique. The study was conducted between January 2013 and April 2017, at 4 hospitals centers in France where both in- and out- patients with MDD were included. Randomization was computer-generated (1:1), with allocation concealment implemented within the e-CRF. The main outcome measure was the percentage of responders 44 days (D44) after the rTMS session. Secondary outcomes were percentage of remitters, Beck Depression Inventory and psychomotor retardation assessed with Salpêtrière retardation rating scale (SRRS) for depression at D14 and D44. The results are presented along with their 95% confidence intervals. Results: 105 patients were randomized and 92 were evaluable with respectively 45 patients in the neuronavigation group and 47 in the standard group. A treatment response was observed for 14 (31.8%) of 44 patients analyzed in the intervention group, and for 16 (35.6%) of 45 patients analyzed in the control group with no statistical difference (relative risk 0.89; 95% confidence interval, [0.50;1.61]). No difference was evidenced for secondary outcomes at D44 whether it concerns remission at D44 (relative risk, 0.82; 95% CI, 0.36 to 1.88), or BDI results (difference in means, 0,01; 95% CI, -3.06 to 3.26), or SRRS results (difference in means, 0.11; 95% CI, -2.42 to 5.02). Similar results were observed at D14. Rates of adverse events were similar in both groups with 23 (47.92 %) and 1 (2.08%) of adverse events and serious adverse events in the neuro-navigation group versus 20 (40.82 %) and 0 (0%) in the standard group. Discussion: This study failed to reproduce previous findings supporting the use of neuro-navigation system to optimize rTMS efficacy. Limitations of this study includes a small sample size and a number of rTMS sessions that may appear substandard in 2020.**

**Trial registration:**

**NCT01677078**

**Funding:**

**Syneika”**

- Adverse event rates are described as "similar," yet no statistical results are reported for this comparison. Statistical tests should be included for consistency.

**Statistical tests were included in the text as required to account for the reviewer’s comment, although we think that for rare events such as serious adverse events, results of statistical tests are not informative.**

**“Rates of adverse events were similar in both groups with 23 (44%) and 1 (2%) of adverse events and serious adverse events in the neuronavigation group versus 20 (38%) and 0 (0%) in the standard group (p-values of 0.55 and 0.49 respectively). Table 3 presents all adverse events.”**

**Thanks to this comment, we also found an inaccuracy in table 3 with the patient with a SAE also counted in AE section. We have corrected this inaccuracy.**

**23 (46%)**

**Please also note that we used for those events Fisher’s exact test and have edited the methods section accordingly.**

**“The primary outcomes as well as all other categorical efficacy outcomes were compared using Chi2. Safety outcomes were analyzed using Fisher’s exact test.”**

Introduction
- The authors aim to "reproduce the findings from Fitzgerald et al." However, their rTMS protocol (20 Hz with 40 pulses per train, 80 trains, inter-train interval of 10 seconds, 110% resting motor threshold, and 10 sessions) differs significantly from that used in Fitzgerald et al. These differences should be explained and acknowledged in the Introduction.

**We thank the reviewer for this conceptual point. While we aimed to reproduce the effect identified by Fitzgerald et al. -the superiority of a neuronavigation system-, we did this in a different setting, by using the parameters that were in use in France at this time. We have added this perspective briefly in the introduction.**

**“We therefore designed this study to reproduce the findings from Fitzgerald et al. (Paul B Fitzgerald et al., 2009) in the French context using a larger sample size and an in-house developed French neuro-navigation system (Syneika; syneika.fr) which takes its originality in the automatic location of the left DLPFC. Thus, the definition of reproducibility includes both the concept of result reproducibility and generalizability, as outlined by Goodman et al. (Goodman et al. 2016). A first objective of this multicenter randomized control trial was to demonstrate the superiority in terms of efficacy over the depressive symptoms of HR-rTMS of the left DLPFC targeted with MRI based neuro-navigation versus the standard localization technique in MDD using the MADRS score.”**

**REFERENCE**

***Goodman SN, Fanelli D, Ioannidis JP. What does research reproducibility mean? Sci Transl Med. 2016 Jun 1;8(341):341ps12. doi: 10.1126/scitranslmed.aaf5027. PMID: 27252173.***

**We have also discussed the differences in the discussion section, following the reviewer’s next comment.**

Methods
- The rationale for using 20 Hz rather than the more common 10 Hz protocol should be provided. While 20 Hz is not inherently problematic, justification for its selection is necessary.

**We thank the reviewer for this request to clarify the parameters that were chosen. We selected 20-Hz stimulation for this protocol. At the time of writing the study, this choice was based on data from Speer et al. (2000) (Speer et al., 2000), which demonstrated that two weeks of daily 20-Hz rTMS over the left prefrontal cortex at 100% of the motor threshold resulted in a sustained increase in regional cerebral blood flow (rCBF) in the bilateral frontal, limbic, and paralimbic regions implicated in depression. This decision was also supported by multiple studies on the efficacy of 20-Hz rTMS administered over the left prefrontal cortex (Fitzgerald et al., 2003; Garcia-Toro et al., 2001; Padberg et al., 1999). We have edited the text accordingly.**

**“The rTMS protocol used a frequency of 20 Hz with 40 pulses per train, 80 trains, and an inter-train interval of 10 seconds, resulting in a total of 3,200 pulses per session. The treatment intensity was set at 110% of the patient's resting motor threshold (RMT) as determined by visual observation. The rationale for using 20 Hz was based on data from Speer et al. (2000) (Speer et al., 2000), which demonstrated that two weeks of daily 20-Hz rTMS over the left prefrontal cortex at 100% of the motor threshold resulted in a sustained increase in regional cerebral blood flow in the bilateral frontal, limbic, and paralimbic regions implicated in depression. This decision was also supported by multiple studies on the efficacy of 20-Hz rTMS administered over the left prefrontal cortex (Fitzgerald et al., 2003; Garcia-Toro et al., 2001; Padberg et al., 1999).”**

**REFERENCES**

***Fitzgerald, P. B., Brown, T. L., Marston, N. A. U., Daskalakis, Z. J., De Castella, A., & Kulkarni, J. (2003). Transcranial Magnetic Stimulation in the Treatment of Depression : A Double-blind, Placebo-Controlled Trial. Archives of General Psychiatry, 60(10), 1002. https://doi.org/10.1001/archpsyc.60.9.1002***

***Garcia-Toro, M., Mayol, A., Arnillas, H., Capllonch, I., Ibarra, O., Crespı́, M., Micó, J., Lafau, O., & Lafuente, L. (2001). Modest adjunctive benefit with transcranial magnetic stimulation in medication-resistant depression. Journal of Affective Disorders, 64(2‑3), 271‑275. https://doi.org/10.1016/S0165-0327(00)00223-8***

***Padberg, F., Zwanzger, P., Thoma, H., Kathmann, N., Haag, C., D. Greenberg, B., Hampel, H., & Möller, H.-J. (1999). Repetitive transcranial magnetic stimulation (rTMS) in pharmacotherapy-refractory major depression : Comparative study of fast, slow and sham rTMS. Psychiatry Research, 88(3), 163‑171. https://doi.org/10.1016/S0165-1781(99)00092-X***

***Speer, A. M., Kimbrell, T. A., Wassermann, E. M., D. Repella, J., Willis, M. W., Herscovitch, P., & Post, R. M. (2000). Opposite effects of high and low frequency rTMS on regional brain activity in depressed patients. Biological Psychiatry, 48(12), 1133‑1141. https://doi.org/10.1016/S0006-3223(00)01065-9***

- Details on statistical methods are insufficient. The authors should specify how they calculated relative risks, absolute risk differences, and 95% CIs, in addition to the Chi-squared tests and t-tests already described.

**We thank the reviewer for this comment. These clarifications have been added and substantially improved the reading of the manuscript.**

**“The primary analysis was a modified intention-to-treat analysis (mITT) where all evaluable patients (with at least a D1 and D14 assessment) were included. Continuous variables are presented as means and standard deviations. Categorical variables are presented as the number of patients in each category and the corresponding percentages. Missing data at D44 were replaced using the Last Observation Carried. Forward approach (this choice, not pre-specified in the protocol, was made before drafting the statistical analysis plan). The primary outcomes as well as all other categorical efficacy outcomes were compared using Chi2 (relative risks, absolute risk differences and 95% confidence intervals were reported). Safety outcomes were analyzed using Fisher’s exact test. Continuous variables were compared using t-tests (mean differences and 95% confidence intervals were reported). Significance level was set out at 0.05. The primary analysis was completed by a sensitivity analysis on the per protocol population. Database was locked on November 2018, statistical analysis plan was drafted on November 2018, and the statistical analysis was completed on December 2018. All analyses were conducted with SAS statistical software, version 9.4 (SAS Institute).”**

Results
- Statistical results for safety data should be reported in greater detail, including relative risks or absolute risk differences, similar to the efficacy outcomes.

**While we appreciate the reviewer’s suggestion to include additional information on adverse events, we believe that it would not provide meaningful insights for serious adverse events, given that only one SAE was reported in the study. Therefore, as we believe the key information is already clearly presented in the numerical estimates in Table 3, we prefer not to modify the text. However, if the editor feels otherwise and requests the inclusion of this information, we would be happy to accommodate that.**

- Given the inclusion of bipolar disorder patients, subgroup analyses should be conducted and reported separately for this population. Previous literature on rTMS for bipolar disorder is limited, and such analyses would add value, particularly in mixed cohorts of major depressive disorder and bipolar disorder patients.

**We thank the reviewer for this comment. While we acknowledge the clinical relevance of the question, we respectfully disagree with the statistical approach suggested. Subgroup analyses carry a risk of alpha inflation and should only be conducted and interpreted under two conditions:**

- **When they are planned a priori**
- **When the results are positive.**

**Since our primary outcome was negative, performing subgroup analyses could lead to false positives. Given this, we believe the previous version of the text adequately addressed this point. However, we have added a clarification to explain why subgroup analyses were not conducted:**

**“A final point that may explain the difference lies in the clinical characteristics of the patients. In our study, we included patients with more heterogeneous types of depression than in Fitzgerald's study. Twenty percent of our patients had bipolar depression, which was an exclusion criterion in the Fitzgerald study. Despite these differences, we chose not to conduct subgroup analyses, as they carry a risk of alpha risk inflation and cannot be reliably interpreted when not planned a priori and/or when the results are negative, as in our case.”**

**Please note that the approach we propose in this response is in line with a comment of reviewer 2.**

Discussion
- The authors state that "the limited number of sessions prevented a more pronounced differentiation between groups." Previous research (e.g., Hsu TW, Neurosci Biobehav Rev, 2024 Jul;162:105704) supports the idea that a greater number of sessions can enhance overall efficacy. The authors are encouraged to elaborate further on this point.

**We thank the reviewer for this comment, and we have now added this reference in our discussion.**

**“Third, the stimulation parameters were noticeably different in both studies. The rTMS treatment was applied at 10Hz with an intensity of 100% of resting motor threshold for a total of 20 sessions per patient in Fitzgerald et al. In comparison, in our study the rTMS treatment was applied at 20Hz with an intensity of 110% of resting motor threshold for a total of 10 sessions per patient. In particular, the limited number of sessions conducted in our study may have influenced the operator-dependent effects in standard positioning and it perhaps prevented a more pronounced differentiation between groups, as observed in the study by Fitzgerald et al. Indeed, previous research (Hsu TW et al., 2024) supports the idea that a greater number of sessions can enhance overall efficacy.”**

**REFERENCE**

***Hsu TW, Yeh TC, Kao YC, Thompson T, Brunoni AR, Carvalho AF, Hsu CW, Tu YK, Liang CS. The dose-effect relationship of six stimulation parameters with rTMS over left DLPFC on treatment-resistant depression: A systematic review and meta-analysis. Neurosci Biobehav Rev. 2024 Jul;162:105704. doi: 10.1016/j.neubiorev.2024.105704. Epub 2024 May 7. PMID: 38723735.***

- The recruitment of ~20% bipolar disorder patients warrants additional discussion. A recent meta-analysis (Hsu CW, Neurosci Biobehav Rev, 2024 Jan;156:105483) suggests that high-frequency stimulation of the left DLPFC may not be optimal for bipolar disorder, potentially impacting overall efficacy. The authors should explore this issue in greater depth.

**We thank the reviewer for this comment, that adds an additional perspective to the comment made before. The text was changed accordingly.**

**A final point that may explain the difference lies in the clinical characteristics of the patients. In our study, we included patients with more heterogeneous types of depression than in Fitzgerald's study. Twenty percent of our patients had bipolar depression, which was an exclusion criterion in the Fitzgerald study. A recent meta-analysis (Hsu CW et al., 2024) suggests that high-frequency stimulation of the left DLPFC may not be optimal for bipolar disorder, potentially impacting overall efficacy.** **Despite these potential differences, we chose not to conduct subgroup analyses, as they carry a risk of alpha risk inflation and cannot be reliably interpreted when not planned a priori and/or when the results are negative, as in our case.**

**REFERENCE**

***Hsu CW, Chou PH, Brunoni AR, Hung KC, Tseng PT, Liang CS, Carvalho AF, Vieta E, Tu YK, Lin PY, Chu CS, Hsu TW, Chen YB, Li CT. Comparing different non-invasive brain stimulation interventions for bipolar depression treatment: A network meta-analysis of randomized controlled trials. Neurosci Biobehav Rev. 2024 Jan;156:105483. doi: 10.1016/j.neubiorev.2023.105483. Epub 2023 Dec 5. PMID: 38056187.***

**We thank the reviewer for these important comments, and hope that we have made all the necessary clarifications. We think that those revisions have substantially improved the paper, that all were really sensible, needed and important for clarifying the messages.**

Reviewer #2: This is a multicenter, double blind, randomized (1:1) controlled trial based on a smaller trial which gave the promise of a response advantage of a new intervention over an established procedure in MDD. The study which was modified intent to treat (MITT) was well designed and well planned.

**We thank the reviewer for this comment.**

Unfortunately it fell short of accrual and the comparative effect expected.

**We thank the reviewer for this comment. While this negative results was disappointing, we believe that it still fits with PLOS criteria for publication even if the trial is negative. As explained in our previous letter, we faced challenges in publishing it. Although the study has several limitations, which we discuss in detail, we believe it remains valuable as outline by the reviewer that the study was “well designed and well planned”. We are aware that PLOS ONE recognizes the importance of publishing negative results and hope that you will consider our appeal of the editorial decision.**

The investigators did well describing the limitations of both the previous small study and their own trial as well.

**We thank the reviewer for this kind comment. As outline in our cover letter, although the study has several limitations, which we discuss in detail, we believe it remains valuable. We really appreciate that the reviewers agree that we did well in describing the limitations.**

The sample size was too small to attempt perhaps some adjusted analysis given the patient characteristics. However, even so, it probably would not have modified the results.

**We thank the reviewer for this comment, and we agree with this approach. This is why we did not perform the subgroup analyses requested by reviewer one as it would carry a risk of type I (and type II error).**

There was mention in the manuscript of a sensitivity study analysis using a per protocol approach with this study which really did not impact the results. However, the protocol included in the supplement was only three pages and lacked much detail, for example, specifically what was the per protocol population. Thus it was difficult to determine the differences between the two strategies.

**Thank you for this comment. This information is actually in the full protocol that was attached (in French):**

**« Il est prévu de réaliser une analyse de sensibilité sur la population en per protocole. Celle-ci se définit comme la population ayant reçu le traitement à l’étude conformément au protocole et ayant bénéficié d’une localisation automatique pour les patients neuronavigués. »**

**The translation (AI generated) is:**

**« A sensitivity analysis is planned for the per-protocol population. This population is defined as those who received the study treatment according to the protocol and who benefited from automatic localization for neuronavigated patients. »**

**We have clarified the text and for further transparency, we have attached the Statistical analysis plan.**

**The SAP reads the same as the protocol:**

**“Rappel : l’analyse de sensibilité en per protocole se définit comme la population ayant reçu le traitement à l’étude conformément au protocole et ayant bénéficié d’une location automatique pour les patients neuronavigués. (cf. protocole)”**

**AI translated:**

**“Reminder: The per-protocol sensitivity analysis is defined as the population who received the study treatment according to the protocol and who benefited from automatic localization for neuronavigated patients (see protocol).”**

**“Missing data at D44 were replaced using the Last Observation Carried. Forward approach (this choice, not pre-specified in the protocol, was made before drafting the statistical analysis plan). The primary outcomes as well as all other categorical outcomes were compared using Chi2. Continuous variables were compared**

**using t-tests. Significance level was set out at 0.05. The primary analysis was completed by a sensitivity analysis on the per protocol population defined as those who received the study treatment according to the protocol and who benefited from automatic localization for neuronavigated patients. Database was locked on November 2018, statistical analysis plan (attached as a supplementary material) was drafted on November 2018, and the statistical analysis was completed on December 2018. All analyses were conducted with SAS statistical software, version 9.4 (SAS Institute).”**

**Please also note that we have corrected a typo in this paragraph by deleting the “.” in “Last Observation Carried. Forward”.**

**We thank the reviewer for these important comments, and hope that we have made all the necessary clarifications. We think that those revisions have substantially improved the paper, that all were really needed and important.**
