# Supplementary material for: Repetitive Transcranial Magnetic Stimulation targeted with MRI based neuro-navigation in major depressive episode: a double-blind, multicenter randomized controlled trial
Source: PLoS One. 2025 May 27;20(5):e0317597. doi: 10.1371/journal.pone.0317597 (PMC12111610; doi:10.1371/journal.pone.0317597)
Supplement: Attachment — Submitted filename: REVISIONS.docx [file pone.0317597.s008.docx]

PONE-D-24-60061R1

Repetitive Transcranial Magnetic Stimulation targeted with MRI based neuro-navigation in major depressive episode: a double-blind, multicenter randomized controlled trial

PLOS ONE

Dear Dr. Naudet,

Thank you for submitting your manuscript to PLOS ONE. After careful consideration, we feel that it has merit but does not fully meet PLOS ONE’s publication criteria as it currently stands. Therefore, we invite you to submit a revised version of the manuscript that addresses the points raised during the review process.

==============================

We would like to express our gratitude to the editor and reviewers for their meticulous review of our manuscript. We have thoroughly addressed all the points that were raised.

==============================

Please submit your revised manuscript by May 08 2025 11:59PM. If you will need more time than this to complete your revisions, please reply to this message or contact the journal office at [plosone@plos.org](mailto:plosone@plos.org). When you're ready to submit your revision, log on to <https://www.editorialmanager.com/pone/> and select the 'Submissions Needing Revision' folder to locate your manuscript file.

Journal Requirements:

When submitting your revision, we need you to address these additional requirements.

1. Please ensure that your manuscript meets PLOS ONE's style requirements, including those for file naming. The PLOS ONE style templates can be found at
<https://journals.plos.org/plosone/s/file?id=wjVg/PLOSOne_formatting_sample_main_body.pdf> and
<https://journals.plos.org/plosone/s/file?id=ba62/PLOSOne_formatting_sample_title_authors_affiliations.pdf>

This has been done.

2. Thank you for providing the following Funding Statement:
"This study was funded by Syneika. The funders had no role in study design, data collection and analysis, decision to publish, or preparation of the manuscript."

We note that one or more of the authors is affiliated with the funding organization, indicating the funder may have had some role in the design, data collection, analysis or preparation of your manuscript for publication; in other words, the funder played an indirect role through the participation of the co-authors.

We are surprised by this comment as no co-author is affiliated with the start-up Syneika. Below is the list of authors along with their respective affiliations:

B Millet,a G Harika-Germaneau,b R Maatoug,a F Naudet,c,d JM Reyman,e V Turmel,e JM Batail,f J Soulabaille,f N Jaafari,b D Drapierf

a: Institut du Cerveau et de la Moelle, UMR 7225, CNRS, INSERM, Sorbonne Université et Département de Psychiatrie Adulte, Groupe Hospitalier Pitié-Salpêtrière, Paris, France.

b: CNRS, Université de Poitiers, Université de Tours, CeRCA, Poitiers, France; Unité de Recherche Clinique Pierre Deniker du Centre Hospitalier Henri Laborit, Poitiers, France.

c: Univ Rennes, CHU Rennes, Inserm, EHESP, Irset (Institut de recherche en santé, environnement et travail) - UMR_S 1085, Rennes, France.

d: Institut Universitaire de France (IUF), Paris, France.

e: Univ Rennes, CHU Rennes, Centre d'investigation Clinique (CIC) Inserm 1414, Rennes University Hospital, Rennes, France.

f: Adult Psychiatry Department, Guillaume-Régnier Hospital, Univ Rennes, CHU Rennes, Centre d'investigation Clinique (CIC) Inserm 1414, Rennes, France.

None of the authors are affiliated with Syneika. One author, BM, has stock options in this startup but receives no salary, as detailed in the COI statement. Despite this transparent disclosure, we confirm that "The funder had no role in study design, data collection and analysis, decision to publish, or preparation of the manuscript."

If the funding organization did not play a role in the study design, data collection and analysis, decision to publish, or preparation of the manuscript and only provided financial support in the form of authors' salaries and/or research materials, please review your statements relating to the author contributions, and ensure you have specifically and accurately indicated the role(s) that these authors had in your study in the Author Contributions section of the online submission form. Please make any necessary amendments directly within this section of the online submission form. Please also update your Funding Statement to include the following statement: “The funder provided support in the form of salaries for authors [insert relevant initials], but did not have any additional role in the study design, data collection and analysis, decision to publish, or preparation of the manuscript. The specific roles of these authors are articulated in the ‘author contributions’ section.”

Such a statement would be innacurate has no author got a salary from Sineyka. We made no change. If you think a change is needed, please clarify the request.

If the funding organization did have an additional role, please state and explain that role within your Funding Statement.

The funding organisation had no additional role.

Please also provide an updated Competing Interests Statement declaring this commercial affiliation along with any other relevant declarations relating to employment, consultancy, patents, products in development, or marketed products, etc.

As none of the authors is employed by Syneika, this change was not made. The relationship between Professor Millet and Dr. Nauczyciel (the spouse of Syneika’s CEO), as well as the role of Dr. Nauczyciel, who is not an author, has been clarified. This aims to address any previously unclear potential conflicts of interest in our study, noting that none of the authors are employed by Syneika. Furthermore, we hope this clarification confirms the accuracy of our statement. We are willing to make further changes if necessary. See our answer to the next question.

Within your Competing Interests Statement, please confirm that this commercial affiliation does not alter your adherence to all PLOS ONE policies on sharing data and materials by including the following statement: "This does not alter our adherence to PLOS ONE policies on sharing data and materials.” (as detailed online in our guide for authors <http://journals.plos.org/plosone/s/competing-interests>). If this adherence statement is not accurate and there are restrictions on sharing of data and/or materials, please state these. Please note that we cannot proceed with consideration of your article until this information has been declared.

We have added the following sentence to the COI disclosure: "This does not alter our adherence to PLOS ONE policies on sharing data and materials." See below :

« All authors have completed the ICMJE uniform disclosure form available at http://www.icmje.org/coi_disclosure.pdf, which can be requested from the corresponding author. The authors declare that they have no conflicts of interest, except for Prof. Millet, who holds stocks in Syneika company. Dr Nauczyciel who is syneika CEO spouse worked on Prof. Millet's team and helped with data acquisition without fulfilling authorship criteria. These potential conflict of interest doe not alter our adherence to PLOS ONE policies on sharing data and materials.»

3. In the online submission form, you indicated that [This study involves human participant data that cannot be publicly shared due to French regulatory constraints, as it includes potentially identifying or sensitive patient information. However, datasets will be made available upon request, subject to the non-opposition of study participants. This non-opposition process may incur minimal additional costs. Data requests can be sent to the Data Privacy Officer at Rennes University Hospital at dpo@chu-rennes.fr.].
All PLOS journals now require all data underlying the findings described in their manuscript to be freely available to other researchers, either 1. In a public repository, 2. Within the manuscript itself, or 3. Uploaded as supplementary information.
This policy applies to all data except where public deposition would breach compliance with the protocol approved by your research ethics board. If your data cannot be made publicly available for ethical or legal reasons (e.g., public availability would compromise patient privacy), please explain your reasons on resubmission and your exemption request will be escalated for approval.

We have clarified the description and request for an exemption as detailled in the following text. Please note that all 3 reviewers consider that we adhere to PLOS policy and therefore that this exemption seems to be adequate (because public availability would compromise patient privacy). We have also clarfied the generic email adress in order to ensure that data is FAIR with a permanent address at Rennes University Hospital. Here is a detailled statement.

This study involves human participant data that cannot be publicly shared due to French regulatory constraints, as it includes potentially identifying or sensitive patient information (i.e. public availability would compromise patient privacy). In the European context, under GDPR it is not possible to share openly this sensitive data, even when it is pseudonimysed. Furthermore, this is an old study where data sharing was not pre-planned in the documents approved by the IRB. This implies that consent for data sharing was not requested when itw as initiated and this was approved by the IRB. Any data request will need to seek non-opposition from study participants, as practiced in the French context. Therefore, datasets will be made available upon request, subject to the non-opposition of study participants. This non-opposition process may incur minimal additional costs. Data requests can be sent to the Data Privacy Officer at Rennes University Hospital at DRI@chu-rennes.fr.

4. We note that the original protocol that you have uploaded as a Supporting Information file contains an institutional logo. As this logo is likely copyrighted, we ask that you please remove it from this file and upload an updated version upon resubmission.

We have edited document and deleted the logo accordingly.

5. Please include captions for your Supporting Information files at the end of your manuscript, and update any in-text citations to match accordingly. Please see our Supporting Information guidelines for more information: <http://journals.plos.org/plosone/s/supporting-information>.

This has been done.

Please review your reference list to ensure that it is complete and correct. If you have cited papers that have been retracted, please include the rationale for doing so in the manuscript text, or remove these references and replace them with relevant current references. Any changes to the reference list should be mentioned in the rebuttal letter that accompanies your revised manuscript. If you need to cite a retracted article, indicate the article’s retracted status in the References list and also include a citation and full reference for the retraction notice.

Done. We see no retracted paper in the list. If you see one, please let us know.

Reviewer #1: Thank you for revising the manuscript and clarifying the diagnostic terminology.

Thank you for this comment.

I would like to address two specific issues:

1. Major Depressive Disorder vs. Major Depressive Episode
In your response, you mention that “major depressive disorder is often used even for bipolar and unipolar depression.” While it is sometimes colloquially used in an imprecise manner, strictly speaking, within the DSM classification system, “Major Depressive Disorder (MDD)” should not include episodes that occur as part of bipolar disorder. In DSM-IV, once a patient has experienced a manic or hypomanic episode, the diagnosis shifts to bipolar disorder, rather than MDD. Therefore, using “major depressive disorder” to encompass both unipolar and bipolar depression is not correct from a nosological standpoint. The more accurate term when referring broadly to depressive episodes—whether unipolar or bipolar—is “major depressive episode.”

We agree with this comment. That’s why we changed the wording to major depressive episode. For clarity purpose, we have again edited a few occurrences that remained in our text.

2. Table 1
You now list three categories: Isolated unipolar depressive episode, Recurrent depressive disorder, and Bipolar disorder. From a diagnostic perspective:
- Isolated unipolar depressive episode typically corresponds to a first or single episode of major depression (i.e., “Major Depressive Disorder, Single Episode” in DSM terminology).
- Recurrent depressive disorder refers to multiple major depressive episodes without a history of mania or hypomania (i.e., “Major Depressive Disorder, Recurrent” in DSM terms).
- Bipolar disorder is used if there has ever been a manic, hypomanic, or mixed episode.
This tripartite classification appears to align more closely with DSM-IV constructs (assuming you distinguish the first unipolar depressive episode from subsequent recurrent unipolar episodes). If your study inclusion criteria specifically required a current major depressive episode, then labeling participants according to these three categories is reasonable, provided you have confirmed that those in the “bipolar disorder” group had a history of mania/hypomania and those in the unipolar groups did not. It would be advisable to ensure that the terms used in Table 1 match official DSM-IV language as closely as possible (e.g., “single-episode major depressive disorder,” “recurrent major depressive disorder,” and “bipolar disorder”).

Many thanks. You are right and we have edited the table accordingly for clarity purpose.

Reviewer #2: My review is unchanged.
XXXXXXXXXXXXXXXXXXXXXXXXXXXXXXXXXXXXXXXXXXXXXXXXXXXXXXXXXXXXXXXXXXXXXXXXXXXXXXXXXX
XXXXXXXXXXXXXXXXXXXXXXXXXXXXXXXXXXXXXXXXXXXXXXXXXXXXXXXXXXXXXXXXXXXXXXXXXXXXXXXXXX

Many thanks, we hope that all comments were addressed. However, these lines of xxxxx are unclear. Please let us know if there was an error.

Reviewer #3: This study compared the efficacy of MRI-based neuronavigation technology and the standard 5 cm technique as targets for high-frequency transcranial magnetic stimulation (rTMS) in the treatment of major depressive disorder (MDD) through a double-blind, multicenter, randomized controlled trial. Although this study did not replicate previous findings supporting the optimization of rTMS efficacy using neuronavigation systems, it still provides valuable clinical evidence for the use of rTMS in treating depression. Overall, the manuscript offers important academic insights, is well-structured, and presents the content in a rigorous manner. While there are some minor issues that need to be addressed, they do not significantly affect the overall quality of the paper. I recommend that the authors make the necessary revisions based on the comments provided, and once these revisions are made, I will be happy to accept the manuscript for publication.
1.In the background section, has a reference been omitted after this sentence?

"In one neuroimaging study of depressed patients, a prefrontal serotonin deficiency at baseline normalized after treatment with rTMS."

Many thanks, we have added the reference for this sentence.

2.This paragraph is repetitive in the discussion section:

"Despite these potential differences, we chose not to conduct subgroup analyses, as they carry a risk of alpha risk inflation and cannot be reliably interpreted when not planned a priori and/or when the results are negative, as in our case. Despite these differences, we chose not to conduct subgroup analyses, as they carry a risk of alpha risk inflation and cannot be reliably interpreted when not planned a priori and/or when the results are negative, as in our case."

Many thanks, this was corrected.
